# Species-Specific Adaptations of Trypanosome Morphology and Motility to the Mammalian Host

**DOI:** 10.1371/journal.ppat.1005448

**Published:** 2016-02-12

**Authors:** Joel L. Bargul, Jamin Jung, Francis A. McOdimba, Collins O. Omogo, Vincent O. Adung’a, Timothy Krüger, Daniel K. Masiga, Markus Engstler

**Affiliations:** 1 Lehrstuhl für Zell- und Entwicklungsbiologie, Biozentrum, Julius-Maximilians-Universität Würzburg, Am Hubland, Würzburg, Germany; 2 Department of Biochemistry, Jomo Kenyatta University of Agriculture and technology, Nairobi, Kenya; 3 Molecular Biology and Bioinformatics Unit, International Centre of Insect Physiology and Ecology, Nairobi, Kenya; 4 Department of Biochemistry and Molecular Biology, Egerton University, Egerton, Kenya; University of Cambridge, UNITED KINGDOM

## Abstract

African trypanosomes thrive in the bloodstream and tissue spaces of a wide range of mammalian hosts. Infections of cattle cause an enormous socio-economic burden in sub-Saharan Africa. A hallmark of the trypanosome lifestyle is the flagellate’s incessant motion. This work details the cell motility behavior of the four livestock-parasites *Trypanosoma vivax*, *T*. *brucei*, *T*. *evansi* and *T*. *congolense*. The trypanosomes feature distinct swimming patterns, speeds and flagellar wave frequencies, although the basic mechanism of flagellar propulsion is conserved, as is shown by extended single flagellar beat analyses. Three-dimensional analyses of the trypanosomes expose a high degree of dynamic pleomorphism, typified by the ‘cellular waveform’. This is a product of the flagellar oscillation, the chirality of the flagellum attachment and the stiffness of the trypanosome cell body. The waveforms are characteristic for each trypanosome species and are influenced by changes of the microenvironment, such as differences in viscosity and the presence of confining obstacles. The distinct cellular waveforms may be reflective of the actual anatomical niches the parasites populate within their mammalian host. *T*. *vivax* displays waveforms optimally aligned to the topology of the bloodstream, while the two subspecies *T*. *brucei* and *T*. *evansi* feature distinct cellular waveforms, both additionally adapted to motion in more confined environments such as tissue spaces. *T*. *congolense* reveals a small and stiff waveform, which makes these parasites weak swimmers and destined for cell adherence in low flow areas of the circulation. Thus, our experiments show that the differential dissemination and annidation of trypanosomes in their mammalian hosts may depend on the distinct swimming capabilities of the parasites.

## Author Summary

African trypanosomes are protist flagellates that are successful parasites in a wide spectrum of hosts. These include humans, where they cause the deadly sleeping sickness, and livestock, where they cause nagana. Nagana has a tremendous negative impact in wide regions of sub-Saharan Africa. The motility of these parasites has been shown to be essential for their survival in all the different environments they inhabit, from the bloodstream of mammals to the gut of the tsetse fly vector. The complex swimming mechanism of trypanosomes has only recently been elucidated in detail, using *Trypanosoma brucei* cells that have been in long term culture. We aimed to characterise and compare the swimming behaviour of several important livestock-infective trypanosome species, isolated directly from the bloodstream. This was done using state of the art microscopy, allowing measurement of their motility with high spatiotemporal resolution. While showing that the basic flagellar propulsion mechanism is the same in all species, we related the trypanosomes motility to their characteristic morphology. We quantified distinct behaviours in the analysed species, which could specifically be manipulated by experimental variations in the physical environment. Importantly, we show that the trypanosome’s morphology and swimming performance could determine the anatomical niche the parasite populates in the host. This would allow differential dissemination of distinct trypanosome species in the mixed infections, which are frequently observed in the wild.

## Introduction

Trypanosomes are extracellular parasites with an exceptionally broad host range [1]. These flagellates thrive in all vertebrate classes and cause severe diseases in man and livestock. Human African trypanosomiasis (HAT), commonly known as sleeping sickness, is a devastating neglected disease of poverty, and trypanosome infestations of livestock cause additional massive economic burden in sub-Saharan Africa. The animal African trypanosomiases (AAT) comprise a set of veterinary diseases, of which the cattle sickness nagana and the equine plague surra are the most prominent. *Trypanosoma vivax* and *T*. *congolense* are the nagana pathogens of cattle, but can also cause disease in other mammals, including sheep, goats, pigs, horses, camels and even dogs. Both species have additionally been identified in a wide range of wild animals, including ruminants and suids, but also lions or hyaenas [2]. *T*. *brucei* is pathogenic to camels, horses and dogs, but is also prevalent in sheep, goats, cattle and pigs as well as in a wide variety of wildlife species. The broad host range is shared by the human sleeping sickness parasite *T*. *b*. *rhodesiense* in east and southern Africa. *T*. *b*. *gambiense* causes HAT in west and central Africa and has been reported only in pigs and some wildlife hosts [3].

Most African trypanosomes are transmitted by the tsetse fly. Due to recent partial loss of the mitochondrial DNA, *T*. *evansi*, now recognised as another subspecies of *T*. *brucei*, is no longer capable of infecting the tsetse fly [4]. Consequently, the prevalence of *T*. *evansi* is no longer restricted to the sub-Saharan tsetse belt. In fact, mechanically transmitted *T*. *evansi* parasites cause surra in horses, mules and cattle not only in Africa, but also throughout large parts of Asia and South America, where the trypanosomes are also found in wild reservoir hosts [5]. Likewise, *T*. *vivax* can be transmitted mechanically and hence, has extended its geographic distribution to South America.

Thus, many trypanosome species are contagious for a wide range of diverse mammals. This distinguishes them from other important parasites, such as *Plasmodium*, which infects only a single genus or even species. *Toxoplasma* infects a wide range of animals, sexual development and oocyte formation, however, occurs only in feline hosts. While those pathogens invade host cells, African trypanosomes prosper extracellularly in the circulation and various tissues. The question arises whether the extraordinary expansion of host range has evolved as a consequence of the extracellular lifestyle.

In fact, all AAT-causing trypanosomes face similar challenges of the mammalian immune system. The defence against host immunity is primarily mediated by sequential expression of antigenically distinct glycosylphosphatidylinositol (GPI)-anchored variable surface glycoprotein (VSG) [6–8], a feature that is known as antigenic variation. The parasites have been shown to exhibit high rates of membrane trafficking [9,10], which enables internalisation of antibody-VSG complexes on the parasites’ surface [11]. Endocytosis in African trypanosomes is localised to the posterior part of the cell, where membrane exchange occurs solely at the flagellar pocket (FP), a specialised flask-shaped invagination. The rate of endocytosis in mammalian stage trypanosomes is exceptionally high [9,10], and has been implicated in survival and parasite infectivity [12,13]. Moreover, membrane transport has been shown to be developmentally regulated [10,14,15] and endocytosis occurs exclusively via clathrin [16–18].

Trypanosome motility has been linked to the parasites´ survival in the mammalian host [11], as well as to successful cell division and development [19–21]. Host antibodies targeted to the VSG coat are shunted to the FP by hydrodynamic drag forces due to the directional motion of the parasite [11].

Significant progress has been made in understanding the basic properties of flagellar dynamics and cellular motility of *T*. *brucei* in culture media [22–24]. However, the physicochemical conditions of culture media differ from that of mammalian blood, their natural environment. In fact, we had previously shown that culture forms of *T*. *brucei* modulate the beat direction of their flagellum in response to purely biomechanical cues [24]. Thus, the microenvironment determines in which direction the trypanosome flagellum beats and hence, in which direction the cell moves.

While these results were conclusive for *T*. *brucei* laboratory strains, the relevance to natural isolates or other trypanosome species has not been shown. Indeed, we lack any quantitative data on trypanosome motility in their natural host environment. Therefore, by using high spatiotemporal resolution microscopy we measured the swimming behaviour of the salivarian trypanosomes in blood from different hosts. High-resolution microscopy allowed the comparative analysis of trypanosome motility at the cellular level, i.e. flagellum-driven motility was detailed to reveal flagellar beat frequency, wavelength and beat-to-beat velocity. In addition, trypanosome swimming in the host’s bloodstream was simulated using two different methods; the first one using methylcellulose to change the fluid environment´s viscosity and the second using polydimethyl siloxane (PDMS)-pillar arrays to mimic swimming between blood cells. Our findings document marked differences in motility patterns and swimming speeds, which are not only species-specific, but may also vary between strains and differentially depend on the micro-environment. We suggest that the type of motion behaviour may contribute to the dissemination and annidation of trypanosomes in their mammalian host.

## Results

### The motility patterns of trypanosomes in blood of different hosts

**Fig 1:**
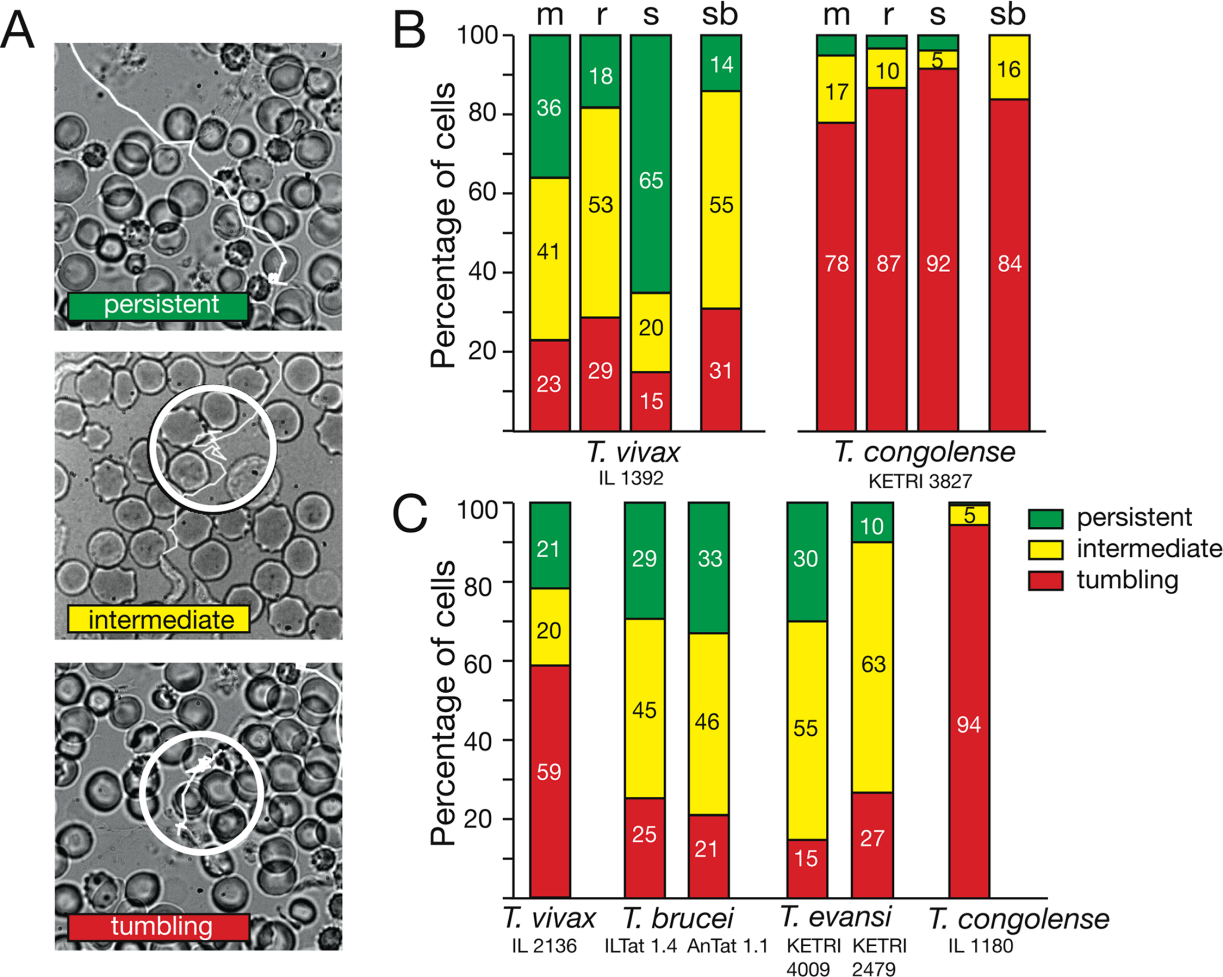
Defined motility patterns characterise the behaviour of trypanosome species in the blood of different hosts. A) Representative examples of three motility types visualised by single cell trajectories. Videos were captured with a frame rate of 500 fps. The swimming paths were traced (white lines), (S1–S5 Videos). Persistent swimmers were defined as cells that swam without a tumbling phase for the duration of a single on-chip recording period (16 s) of the high speed camera. Within this time, intermediate swimmers exhibited at least one tumbling phase. This is a period of two seconds or longer, in which the cells do not leave a circular area with a diameter of 25 μm (white circle). Tumbling cells stay within this area for the complete observation period of 16 s. B) Comparative motility analysis of *T*. *vivax* and *T*. *congolense* in blood freshly harvested from infected mouse (m), rat (r) and sheep (s). The host animals revealed a comparable parasitaemia in the range of 10^7^ trypanosomes/ml. For comparison, parasites grown in mice were incubated in sheep blood (sb). C) Comparison of the locomotion behaviour of four trypanosome species in mouse blood. All parasites were isolated from infected mice and analysed in fresh neat mouse blood. At least 300 cells were analysed per infection and the motility types were scored as shown in (A).

We compared the swimming behaviour of the animal parasites *T*. *vivax*, *T*. *brucei*, *T*. *evansi* and *T*. *congolense* in fresh wet blood films. Trypanosomes were classified by the swimming pattern they exhibit on time scales of seconds to tens of seconds. During these periods, the cells can swim persistently in one direction and cross spaces of several hundred micrometres, but they can also exhibit periods of non-directed movement, in which they do not move much further than their body length, i.e. around 20 microns. This behaviour is reminiscent of the bacterial “run and tumble” motion and trypanosomes have thus been classified as persistent or tumbling swimmers. We have termed cells alternating between the two modes intermediate or switching swimmers (Fig 1A) [22,23]. The motion patterns show a clear difference between *T*. *congolense* on one hand, with 78% to 92% tumbling cells (n ≥ 300), and *T*. *vivax*, *T*. *brucei* and *T*. *evansi* on the other hand. Interestingly, we also observed differences between strains of the same species. *T*. *vivax* strain IL 1392 harvested from infected mice revealed just 23% tumbling cells, while strain IL 2136 showed 63% tumblers in mouse blood (Fig 1B and 1C). When *T*. *vivax* IL 1392 was isolated from sheep, 65% of cells were persistent swimmers, while only 18% of parasites were persistently moving when grown in rat (Fig 1B). *T*. *evansi* grown in mice also revealed clear strain-specific differences (Fig 1C).

In view of the remarkable variability, we devised a series of experiments to unravel the distinguishing basis of the different motion pattern of trypanosomes, both on the population and the single cell level.

**Fig 2:**
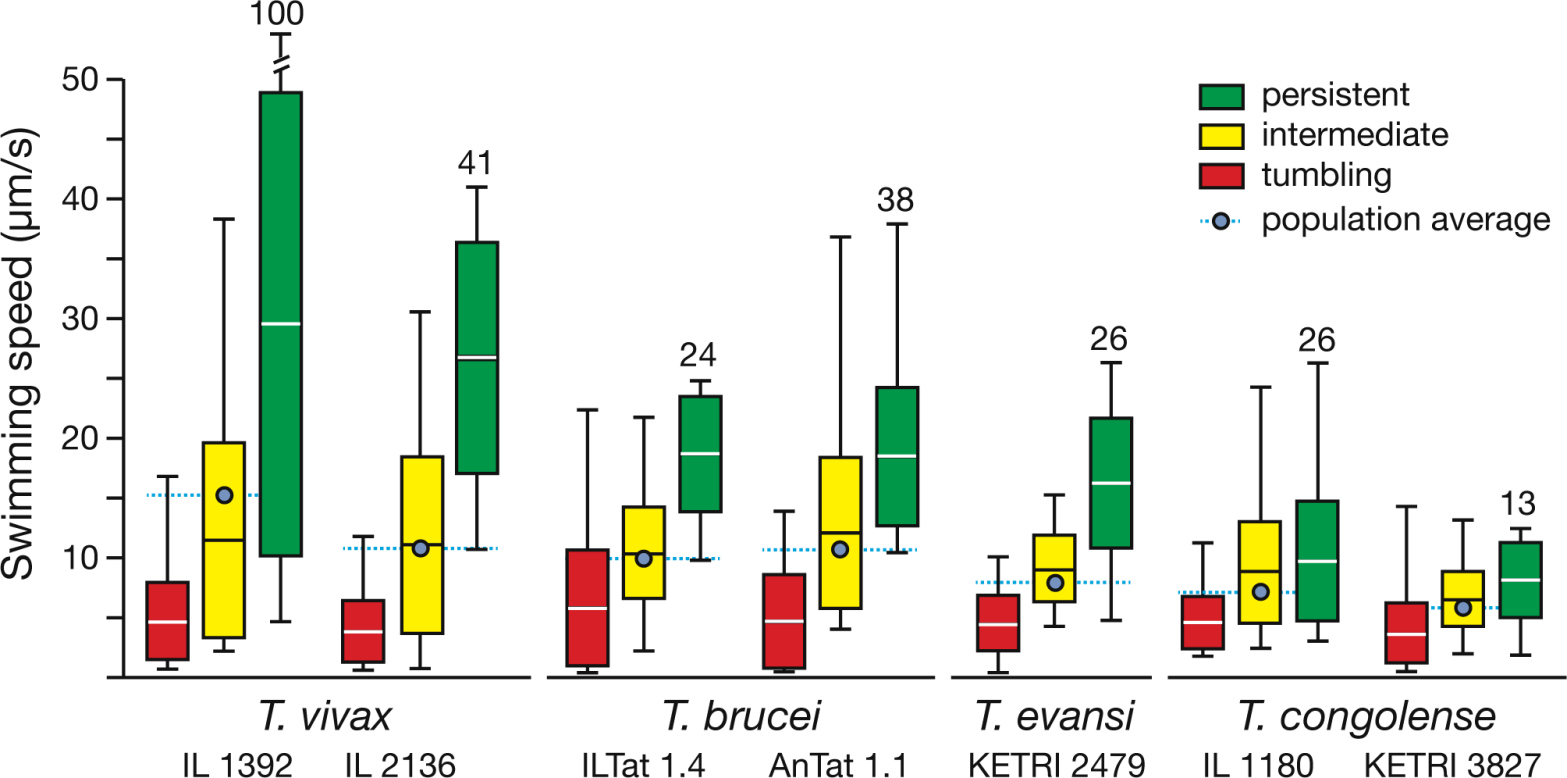
Trypanosome velocities in mouse blood. Each bar represents the analysis of 100 high-speed videos (500 fps, 16 s recording time). The bars present the mean swimming speed +/- SD. Black lines depict the individual maximum and minimum speeds recorded. The maximum speeds for persistent swimmers are annotated. The average population swimming speed of a species was calculated from all 300 trajectories and is marked by the blue dot.

First, we directly compared the swimming speeds of the different parasites. High-resolution microscopy allows to measure translocations of single cells with 2 ms accuracy [24]. This temporal resolution allowed us to separate the translational swimming phases from even brief tumbling phases. The measurements yielded maximum and minimum velocities, and also the average swimming speeds for tumbling, intermediate and persistent subpopulations (n = 100 each) (Fig 2). For all trypanosomes analysed, the mean speeds of persistent swimmers were at least twice as high as those of tumbling cells. Obviously, the actual swimming stretches of tumblers stay in the defined 25 μm region (Fig 1). Nevertheless, the maximum speeds measured in these short time periods can reach those of persistently swimming cells. The trypanosomes can reach these high velocities and halt again so rapidly, because their physical environment is characterised by extremely low Reynolds numbers and inertia is irrelevant. Therefore, all accelerating or decelerating forces produce immediate reactions of the cells [25]. The speeds of the intermediates were in between, showing that the average swimming speed correlates with the overall classification of motility patterns. Although the mechanism of translocation allows many cells to reach high swimming speeds, at least on a short time scale, the lower average speeds indicate that many cells do not accelerate equivalently. These results can only be explained by the detailed analysis of the swimming mechanism in the millisecond timescale. A remarkable result was the maximum swimming speed of a subset of persistent *T*. *vivax* cells of over 100 μm/s (Fig 2). These cells represented about 1% of the population and were characterised by a very straight appearance and slim swimming trajectory (see below).

**Fig 3:**
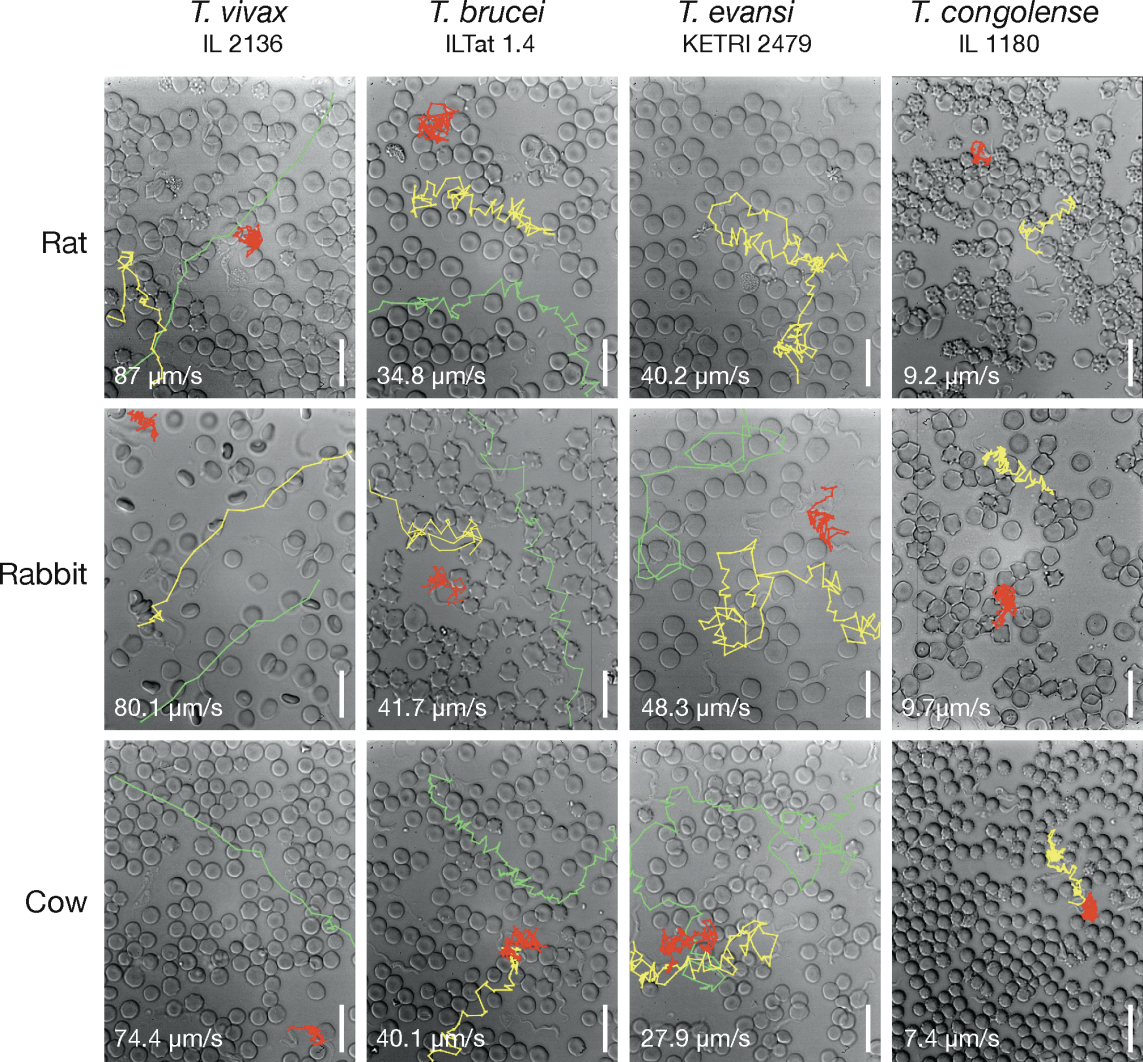
Representative examples of trypanosomes swimming in blood from different host animals. The images are stills of the corresponding S6 Video. Videos were captured with a frame rate of 500 fps. The swimming paths were traced using the Fiji plugin MTrackJ [26]. The trajectories of one example each of tumbling (red), intermediate (yellow) and persistent (green) swimmers are shown. The numbers are maximum speeds (n = 20).

In the next step, we compared the effect of whole blood from different mammals on the motion of the parasites. The trypanosomes were incubated in blood freshly drawn from rat, rabbit and cow (Fig 3). These experiments were done under controlled conditions with parasites from the same mouse infection. In this way, we were able to observe the immediate impact of different blood sources on trypanosome swimming. Fig 3 reveals representative examples of tumbling, intermediate and persistent swimmers in blood from different sources, including the maximum speeds recorded (n = 20). The microscopic observation of the parasites and the measurement of swimming speeds did not reveal any obvious differences in the motion behaviour of the parasites in blood from diverse sources.

Having measured the swimming capacity on the population level, we subsequently proceeded to single cell analyses of motility and morphology to elucidate the potential structural basis for the different swimming behaviours.

### High-resolution motility analysis reveals specific cellular waveforms

**Fig 4:**
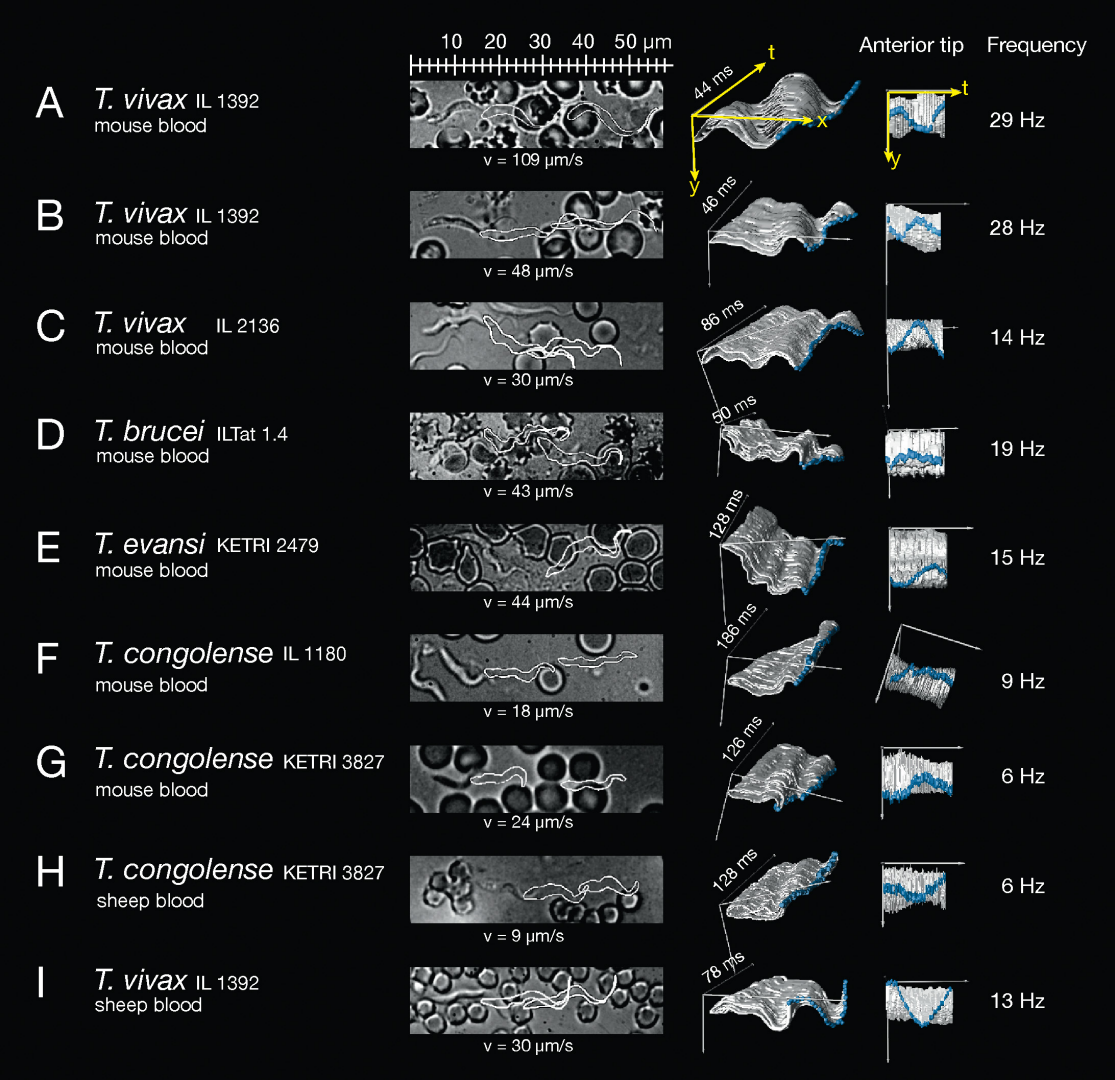
High-resolution single cell analysis of persistently swimming trypanosomes. The images on the left are single frames of the corresponding S8–S16 Videos, showing the cells at the beginning of an analysed flagellar beat, while two later positions of the parasites are shown as white outlines to depict the trajectory of the cell. The speed (v) is the highest average velocity reached during the video sequence analysed. The panel to the right presents the trypanosome outlines from each recorded frame for one complete flagellar beat. The successive image frames were stacked along the time axis in a three-dimensional surface representation. This allows the visualisation of the cellular waveform produced by the beating flagellum and the attached cell body. The anterior tip of the flagellum is marked in blue. The duration of the complete flagellar beat is annotated on the t-axis (ms). The right panel reveals the same 3D-surface representation, however, turned to view on to the anterior tip of the flagellum (along the x-axis of movement). This illustrates the sinusoidal oscillation of the flagellar tip, starting the travelling wave running along the body to the posterior end. The average frequency (Hz) of the flagellar beats of the analysed video is given on the right (see Materials and Methods). A) A representative slim waveform of *T*. *vivax* IL 1392 swimming forward with continuous tip-to-base beats in a wet blood film from the mouse (S8 Video). This waveform represents a small subset (1%) within the parasite population and swims significantly faster than cells exhibiting normal waveforms. B) Normal waveform of *T*. *vivax* IL 1392 in mouse blood from S9 Video, swimming with a frequency similar to that of the fast waveform in A) but reaching less than half the speed. C) *T*. *vivax* IL 2136 in mouse blood reveals slower motion when compared to the IL 1392 strain (S10 Video). D) *T*. *brucei* ILTat 1.4 swims with a lower beat frequency than normal waveform *T*. *vivax* cells in B) but reaches similar speeds (S11 Video). E) *T*. *evansi* KETRI 2479 swimming in mouse blood (S12 Video), with an intermediate beat frequency. The *T*. *evansi* waveform typically appears more elastic and curly when compared to the other trypanosomes, propelling the cells to relatively high velocities along curvy trajectories. F) *T*. *congolense* IL 1180 swimming with characteristic low frequency beats (S13 Video). The anterior tip shows far shallower oscillations due to the missing free anterior part of the flagellum and the stiff cell body causes a waveform clearly less effective for propulsion than that of the other species. Nevertheless the cell shows persistent forward swimming periods with speeds of around 20 μm/s. G) *T*. *congolense* KETRI 3827 swimming in mouse blood (S14 Video), with a lower frequency than strain IL 1180, but with an apparently more effective waveform. H) *T*. *congolense* KETRI 3827 from infected sheep (S15 Video) beat with the same frequency as in mouse blood, but with significantly reduced forward swimming speed, showing the influence of the specific host environment. I) *T*. *vivax* IL 1392 from infected sheep (S16 Video) swim slower and with decreased flagellar beat frequency when compared to cells in mouse blood (B).

High-speed analyses on the single cell level and with single flagellar beat accuracy documented the immediate dependence of cell locomotion on flagellar oscillation (Fig 4). Persistently forward moving cells of all four species exhibited successive flagellar beats originating at the anterior tip of the free flagellum and generating a tip-to-base wave running along the cell body to the base at the flagellar pocket (exemplified in the annotated S7 Video of the *T*. *vivax* cell in Fig 4A). Each persistently swimming cell exhibited tip-to-base beating with a fairly constant frequency. Interestingly, cells with the same beat frequency were observed to swim with significantly different speeds.

In the case of the two *T*. *vivax* strains analysed, about 1% of the population exhibited a slim waveform, i.e. a markedly straighter and thinner appearance, as revealed by the microscopic observation of several thousand parasites. The slim trypanosomes were characterised by the flagellum forming just one wavelength along the entire length axis of the parasite. Thus, the flagellar wave dominates the shape of the cell, thereby producing very effective persistent swimmers that reached high swimming speeds of over 100 μm/s (Fig 2; single cell analysis in Fig 4A). The majority of *T*. *vivax* parasites, however, showed a more flexible form of the flagellar wave, resulting in average speeds of around 30–40 μm/s (single cell analysis in Fig 4B). In both conformations the flagella beat with almost identical average frequencies. The slim single-wave type of *T*. *vivax* represented an ideal oscillatory conformation of the complete cell, resulting in a highly propulsive travelling wave along the cell body.

As in trypanosomes the flagellum is uniquely attached to the cell body, the flagellar oscillation continuously modulates the shape of the cell, rendering it virtually impossible to deduce a static morphological picture of the swimming parasite. We therefore suggest the term “cellular waveform”, which is the product of the flagellar wave, the chirality of the flagellum attachment and the degree of cell stiffness. The overall appearance of the cellular waveform is characteristic for each trypanosome species, but can be modulated in a strain-specific manner.

It is to be noted, that the *T*. *vivax* slim waveforms readily convert briefly into the normal waveform, when hindered in free movement (S17 Video), whereas the reverse change of the unobstructed normal waveform converting into the slim waveform has not been observed. In fact, the slim waveform is not necessarily longer or slimmer than other trypanosome cells but only appears so in motion, because of its straight cellular waveform and high speed. This fact, together with the occurrence before peak parasitaemia, argue against this form being the late stationary developmental stage identified in infected mice, which additionally has a sub-terminally located kinetoplast [27]. Thus, two different subpopulations of *T*. *vivax* are present during infections, which do not represent distinct morphotypes but different cellular waveforms, once more underlining that motion and morphology are interlaced in trypanosomes.

*T*. *brucei* and *T*. *evansi* reveal comparable swimming patterns, velocities and flagellar beat frequencies (Figs 1C, 2, 4D and 4E). This is expected, as *T*. *evansi* most likely represents a mutant of *T*. *brucei*, adapted to mechanical transmission. Interestingly, however, the cellular waveform distinguishes both parasites. Fig 4E shows a typical example of a persistent forward swimming *T*. *evansi* cell. The parasites revealed a more flexible appearance with several short wavelengths of the flagellum running along a rather elastic cell body. This produced the characteristic ‘curly’ waveforms of *T*. *evansi* and the resulting smooth, snake-like forward movement, which was observed to be strongly influenced by mechanical interaction with the environment (S12 Video, see single beat analysis below).

The proportion of persistent swimmers in *T*. *congolense* populations was comparatively low. Nevertheless, the cells were able to swim forward, albeit for shorter periods, with speeds around and significantly above 20 μm/s (Fig 4F and 4G). The oscillation of the anterior flagellar tip was not as pronounced as in other species, as there is no free part of the flagellum. Thus, the beating resulted in weaker oscillations and altogether a less propulsive cellular waveform of the short rigid cell.

Interestingly, *T*. *vivax* IL 1392 and *T*. *congolense* KETRI 3827 showed a marked reduction of swimming speed in sheep blood compared to mouse blood (Fig 4H and 4I). This was not due to any adherent interaction with the blood cells (S15 and S16 Videos, see [28]). We have specifically analysed freely swimming parasites in this work. Thus, the host environment influences the swimming performance of the two most different trypanosome swimmers in a comparable way.

### Trypanosomes differ in flagellum chirality and cell flexibility

**Fig 5:**
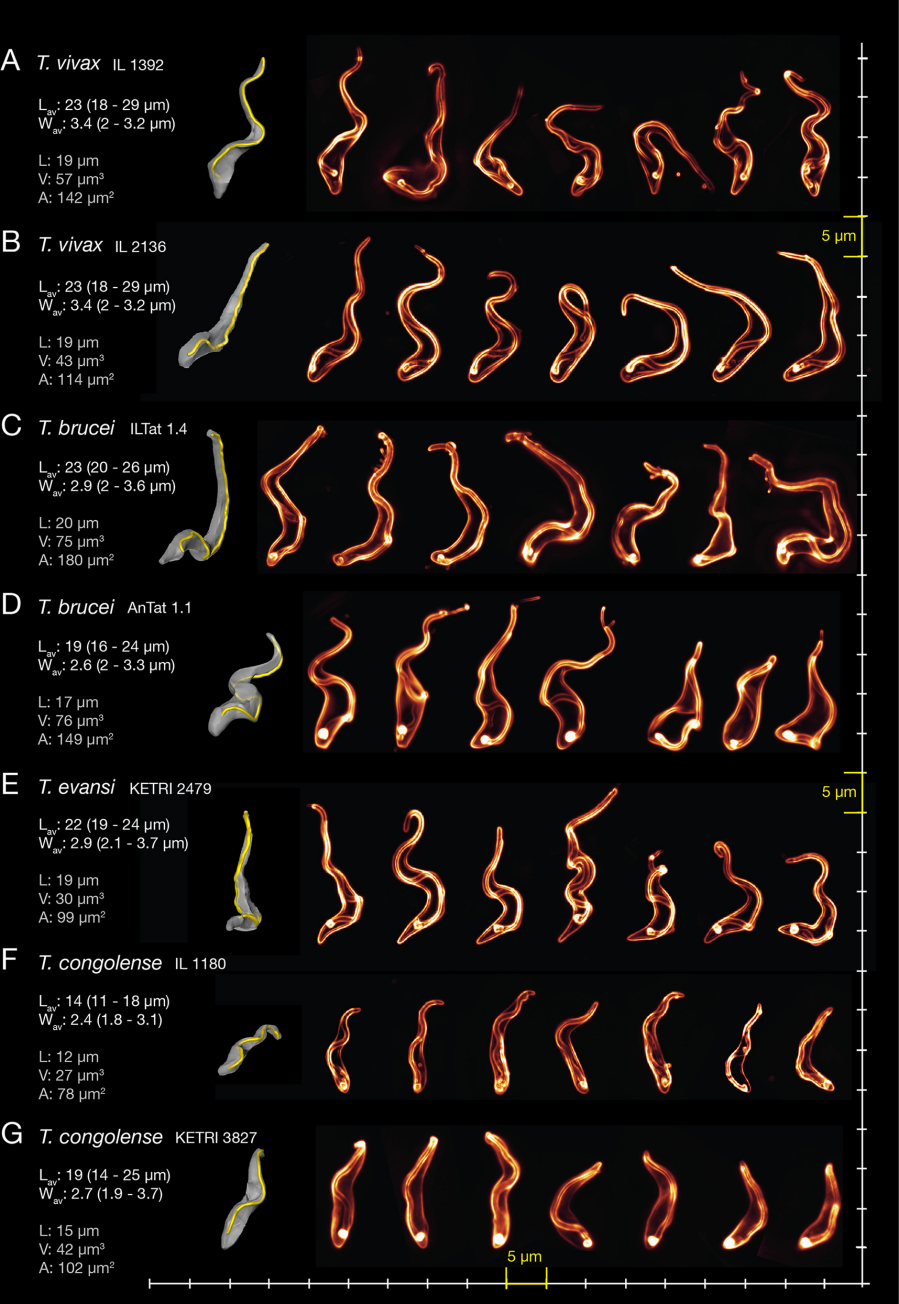
Three-dimensional modelling of trypanosome cells reveals characteristic morphologies correlating to the species-specific motility behaviour. The parasites were harvested from infected mice and the cell surface was fluorescently labelled with sulfo-NHS dyes. On the left of each panel representative surface-rendered models of fluorescently surface-labelled trypanosome cells are shown. The fluorescence labelling allows the simultaneous visualisation of the cell body and the flagellar membrane. The complete trace of the flagellum (yellow) attached along the cell body (grey) is shown in the three-dimensional representation. The model was orientated to show the view onto the posterior tip, allowing to evaluate the course of the flagellar attachment along the cell body, starting at the exit from the flagellar pocket at the top of the cell and tracing it towards the anterior end. A selection of 3D-models is presented to illustrate the variety of shapes the trypanosomes adopt, based on their cellular waveform. The numbers present the average length of the trypanosomes L_av_ (n = 100) and the average maximum width of the cells W_av_ (n = 100) as measured in video stills (see Fig 1). The actual length L, volume V and cells surface area A of the surface-rendered models are given below in grey letters. The first cell in each row was chosen for surface-modelling. (A) Examples of normal waveform *T*. *vivax* IL 1392 cells. The flagellum is attached in a shallow 180° right hand turn. (B) *T*. *vivax* IL 2136 normal waveforms. The flagellum exhibits a similar course to IL 1392 running along a slightly stiffer cell body. (C) The *T*. *brucei* ILTat 1.4 strain typically shows a more prominent 180° right handed turn around the posterior third of the cell body. (D) The pleomorphic *T*. *brucei* AnTat 1.1 strain slender and stumpy forms. The three cells on the right are short stumpy trypanosomes that are characterised by the absence of a free part of the flagellum. The slender forms show a similar 180° right hand turn when compared to ILTat 1.4 in C). Morphometric data are for the slender stage (E) *T*. *evansi* reveals a curlier waveform than *T*. *brucei* while the flagellum turns completely (360°) around the cell body.(F) *T*. *congolense* IL 1180 cells are small with a stiff cell body and a flagellum running along it in a relatively straight course. (G) *T*. *congolense* KETRI 3827 is larger than the IL 1180 strain, but reveals the same characteristic waveform.

In order to better understand the cellular waveform as the basis for the motile behaviour of different trypanosome species and strains, we analysed some morphological key-parameters, such as the flagellar attachment, the cell dimensions and the apparent cell flexibility. We fluorescently labelled the cell surface of *T*. *vivax*, *T*. *brucei*, *T*. *evansi* and *T*. *congolense* and recorded a several hundred three-dimensional microscopic image-stacks, in order to produce three-dimensional representations of the cells (Fig 5). In all trypanosomes analysed the flagellum was closely attached to the cell body and did not constitute part of an elastic undulating membrane.

The two *T*. *vivax* strains showed a rather rigid conformation of the cell body. The flagellum attaches in a right-handed path around the cell. The turn reaches around 180°, but the path is less curved as in *T*. *brucei*, possibly due to a greater stiffness of the cell body (Fig 5A and 5B).

The morphology of the cell culture-adapted *T*. *brucei* strain MITat 1.6 had previously been described as an on average s-shaped cell body with an attached flagellum running from the flagellar pocket in a left hand 180° turn around the cell and continuing along a thinning body to the very flexible anterior end [24]. In comparison, we found that the cells of the *T*. *brucei* ILTat 1.4 strain analysed here, generally were larger and showed a longer and thinner appearance. Interestingly, the flagellum revealed mirrored chirality, i.e. it followed a right-handed turn of 180° around the cell body, from the emergence at the flagellar pocket to the free anterior end (Fig 5C). Analysis of the pleomorphic *T*. *brucei* AnTat 1.1 strain then showed the same right-handed chirality (Fig 5D). We conclude that in *T*. *brucei* the flagellum generally follows a 180° path around the cell and contributes to the characteristic cellular waveform, which however, is not influenced by the rotational direction of flagellum attachment.

The cell body of *T*. *evansi* represents the most flexible of the cell types analysed here. The flagellum followed a full 360° right-handed path around the cell body from posterior to anterior (Fig 5E). These features give *T*. *evansi* cells their typically curled appearance, which distinguishes them from *T*. *brucei* and contributes to their characteristic cellular waveform.

*T*. *congolense* cells were typically the smallest and the cell body seemed the least flexible (Fig 5F and 5G). The flagellum followed a relatively straight path along the cell body, the flagellar turn still producing asymmetry, but usually far less than the 180° half turns of *T*. *brucei* or the full turn *T*. *evansi* (Fig 5C–5E). There was hardly any free part of the anterior end of the flagellum visible. This explains the relatively ineffective force generation of the small and stiff cell bodies of *T*. *congolense*, making these parasites the slowest swimmers. Interestingly, the stumpy form of *T*. *brucei* shares similar characteristics with *T*. *congolense*, a reduced cell length and the lack a long free anterior part of the flagellum, although the cellular waveform is more flexible (Fig 5D, right). Like *T*. *congolense*, the stumpy cells are slow swimmers. In fact, most cells in a stumpy trypanosome population tumble, and only at higher viscosities the parasites swim for short periods. (S18 Video).

In conclusion, we can clearly discern characteristic 3D-morphometric features of different trypanosomes that contribute to their cellular waveform and hence, influence the movement of the parasites.

### Extended single beat analyses reveal millisecond changes in flagellar activity

**Fig 6:**
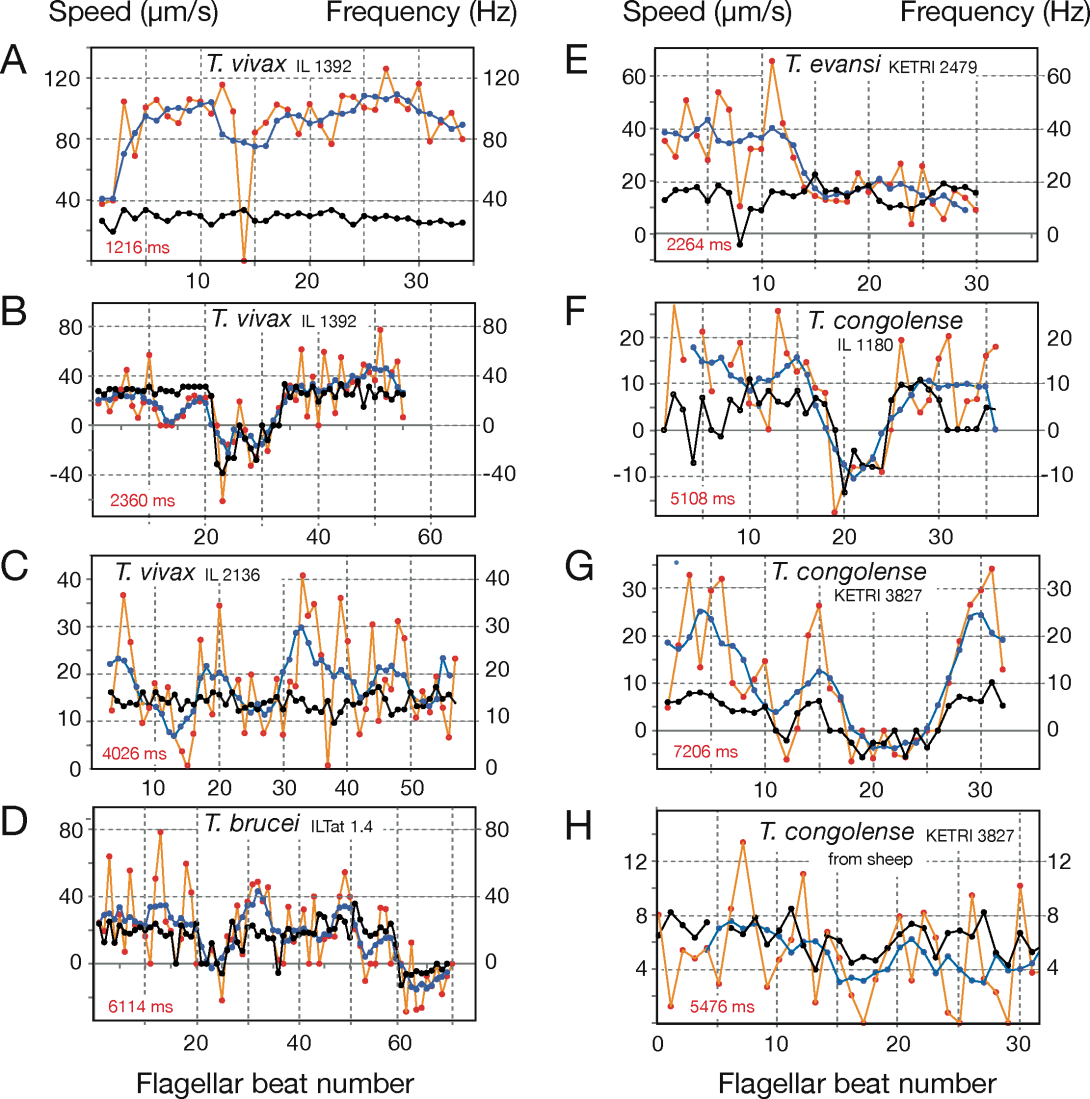
Extended single flagellar beat analysis of trypanosome motility in wet mouse blood. The graphs plot the velocities produced by single, consecutive flagellar beats (red dots), together with the beat frequency (black dots). The velocities, which were derived from measurement of the translocation of the posterior tip after each flagellar beat (white marks in S8–S16 Videos), are averaged over 5 beats to generate the average directional speeds (blue dots). The red number is the overall observation period (ms). A) Fast type of a *T*. *vivax* IL 1392 persistent swimmer with a slim waveform (S8 Video), showing a 30 ms—stop upon mechanical hindrance. The flagellar tip directly hit an erythrocyte, whereupon the trypanosome stopped without flagellar beating being interrupted. Translocation resumed with the next flagellar beat. Note that neither beat frequency nor average velocity (blue line) were markedly changed by this stop. B) Example of a *T*. *vivax* IL 1392 normal waveform swimmer revealing a short (< 1 second) period characterised by several base-to-tip beats, resulting in backward movement, i.e. negative speed (S9 Video). C) *T*. *vivax* IL 2136 exhibited lower beat frequencies than the IL 1392 strain (S10 Video). D) An intermediate *T*. *brucei* ILTat 1.4 swimmer exhibited two persistent swimming stretches interrupted by a short tumbling phase. This was followed by a period of beat reversal and backward swimming (S11 Video). E) A persistently swimming *T*. *evansi* parasite (S12 Video) reducing velocity by half, while the flagellar beat frequency remained in a constant range. (F) *T*. *congolense* IL 1180 showed a short period of rather fast backward motion (S13 Video). (G) *T*. *congolense* KETRI 3827 switching between fast forward and slow backward movement (S14 Video). H) *T*. *congolense* KETRI 3827 isolated from infected sheep revealing persistent slow forward motion (S15 Video).

So far, we had analysed regular persistent forward movement and obtained an overview of the various morphotypes and cellular waveforms that could be correlated to the typical swimming performance of different trypanosome species. Using microscopic datasets of sufficient spatiotemporal resolution, we now analysed millisecond variations of swimming patterns, which were observed to happen with or without environmental influences. In view of the variability documented in the above, we asked if the basic swimming pattern (i.e. forward, backward and tumbling motion) were produced by the same mechanism on the scale of single flagellar beats in all trypanosome species analysed. Trajectories of swimmers were followed in wet blood films and the cells´ translocation speed was measured for each flagellar beat (Fig 6), as defined in the analysis shown in Fig 4. The full trajectories are shown in S8–S16 Videos. These analyses detailed the responses of individual cells to the forces produced by the flagellar waves in the surrounding blood serum, and to the obstacles encountered therein.

As shown above, two subpopulations of *T*. *vivax* exist in freshly drawn mouse blood, which are characterised by distinct cellular waveforms. The cell in Fig 6A (S8 Video) represents the slim waveform (about 1% of all cells) and moved persistently forward with an average speed of 95 μm/s and a flagellar beat frequency of 29 Hz (Fig 6A). Notably, when the anterior tip of the flagellum hit an obstacle frontally, as exemplified by a red blood cell, the cell came to an immediate halt (beat 14, Fig 6A, S8 Video). The beat frequency did not change, i.e. regular flagellar oscillation did not stop, and with the next beat, the cell immediately continued its forward movement with the same speed.

At the high spatiotemporal resolution used here, the two-dimensional speed measurements using a single reference point automatically produce fluctuations, depending mainly on the rotation of the trypanosomes and their three-dimensional helical path. Therefore, the single beat measurements (Fig 6, orange lines) are overlaid with averaged velocities (average of five successive beats, blue lines). Both speeds are shown to detect short-term changes in movement that were masked by averaging (i.e. beat 14 in Fig 6A), underlining the importance of high temporal resolution.

The second *T*. *vivax* example shows the predominant slower normal waveform (Fig 6B, S9 Video). The average speed of this persistent swimmer was 30 μm/s, with a flagellar beat frequency of 27 Hz. The parasite swam through a group of blood cells and came to a brief halt during beats 12–14. In this case also, the beating frequency did not change, but the flagellar wave was not propagated along the cell body to the posterior end, rendering these tip-to-base beats ineffective. The trypanosome then moved forward again for several beats before it started to produce base-to-tip waves (beat 22). Flagellum beat reversal has long been observed in trypanosomatids, but its biological relevance is still not clear [29–32,24].

In this example, the reversal of the flagellar wave, initiated by base-to-tip beats, caused an immediate reversal of translocation. This reversal led to a tumbling phase of around 800 ms (beats 22–33), during which the cell produced seven base-to-tip beats. The reverse waves were mostly incomplete and interrupted by short periods without effective force generating flagellar movements (shown with a frequency value of 0 Hz). Therefore there was only a slight backward translocation of the cell. After the tumbling phase, the cell resumed flagellar beating, immediately producing full tip-to-base waves with the same frequency as before the tumbling period, effectively propelling the cell forwards again at average speeds of around 40 μm/s. This brief tumbling phase changed the parasite’s forward swimming direction through the reorientation of the flexible anterior part of the flagellum. The tip-to base waves running along the cell body cause the cell to rotate and break out in a new direction that is targeted by the flagellar tip, in this case a course change of about 30 degrees (S9 Video).

The analysis in Fig 6C shows a normal waveform T. vivax IL2136 cell beating persistently with an average frequency of 14 Hz (S10 Video). Despite the relatively constant beat frequency, the average speed fluctuates around a mean of 18 μm/s. This is partly due to mechanical interactions, as at around beat 15 the anterior tip of the flagellum collides with another trypanosome slowing forward movement. On the other hand, around beats 33 and 47 higher speeds are measured, as the flagellum interacts laterally on both sides with red blood cells and other obstacles. Here the resistive force produced as the flagellum wriggles through confined spaces accelerates the cell.

The analysis in Fig 6D of a *T*. *brucei* cell swimming through relatively dense red blood cells, shows persistent swimming stretches with an average speed of 24 μm/s and a beat frequency of 18 Hz, interrupted by several short stops which are caused by discontinued flagellar beating without obvious mechanical hindrance. Beat 61 and several following are base-to-tip beats with an average frequency of 5 Hz which effectively propel the cell backwards at average speeds of up to 15 μm/s through an obstacle-free area.

The single beat analysis of a *T*. *evansi* parasite details the impact of the characteristic cellular waveform with its elastic appearance on trypanosome propulsion (S12 Video). In the first part of the trajectory, the cell moved persistently with an average speed of 42 μm/s and a beat frequency of 15 Hz (Fig 6E). During this movement there was one incomplete base-to-tip wave (beat 8). This caused the cell to decelerate and reoriented the anterior tip of the flagellum with the next tip-to-base beat. With the following tip-to-base waves, the cell accelerated back to higher swimming speeds in the altered direction. In its further path, the trypanosome experienced significant resistance from relatively densely packed erythrocytes. While the cell swam in confinement between blood cells, it retained a relatively constant beat frequency, but was routed in a circular path with reduced swimming speed.

Although the general motility pattern of *T*. *congolense* differs greatly from that of *T*. *vivax* (Fig 1B and 1C), we found that the underlying mechanism of bi-directional flagellar beating is conserved in all trypanosomes analysed.

Fig 6F shows two stretches of a *T*. *congolense* IL1180 cell swimming persistently forward, interrupted by a backwards swimming phase. Interestingly, in both directions the cell reaches approximately the same speeds and flagellar beat frequencies. Fig 6G exemplifies a *T*. *congolense* KETRI 3827 cell that swam forward with an average speed of 17 μm/s and a flagellar beat frequency of 6 Hz. The cell then reversed its beat direction, causing an immediate backward propulsion, accelerated forward again using three tip-to-base beats and then entered a tumbling phase of about 3 seconds, consisting of five base-to-tip beats and millisecond periods without any flagellar movement. After this phase, the cell resumed forward swimming with basically the same frequency and speed as before tumbling. The last example shows a *T*. *congolense* KETRI 3827 cell swimming persistently forwards in sheep blood at reduced speeds as compared to this species swimming in mouse blood (Fig 6H, S15 Video)

The above single cell analyses underlined that the principle of flagellar propulsion is conserved in all studied trypanosome species. The marked differences shown for short- and medium-term swimming behaviours is explained by the dynamic and morphological characteristics of a given cell, i.e. its cellular waveform that is controlled by beat frequency, length and amplitude of the flagellum and the direction of flagellar waves running along the cell body.

### The influence of viscosity and confinement on trypanosome motility

We have shown that trypanosome motility can be influenced by the direct physical environment, in other words, by the amount and distribution of resistive force on the distorting flagellum and cell body [24]. In the wet blood films used for the characterisation of swimming patterns on the single cell and population level, the cells move in an environment with random concentration and distribution of blood cells. This is different from the circulation, as blood is a self-stirring fluid. Therefore, we used systems that more closely mimic certain aspects of the *in vivo* environment of bloodstream form trypanosomes.

Therefore, we adjusted the viscosity of blood samples by addition of methylcellulose. A 0.2% methylcellulose solution has a kinematic viscosity (2.5 mPa·s) around 150% higher than that of serum. The addition of methylcellulose to a concentration of 0.4% results in a viscosity (5.2 mPa·s) comparable to fast flowing normal whole blood (4–5 mPa·s) and the viscosity of 0.6% methylcellulose (25 mPa·s) is in the upper range of reported blood viscosity. Values above that (0.8% methylcellulose) are in the lower range of oil viscosities at room temperature. For each condition the trajectories of 300 parasites were analysed and scored as in Fig 1A.

**Fig 7:**
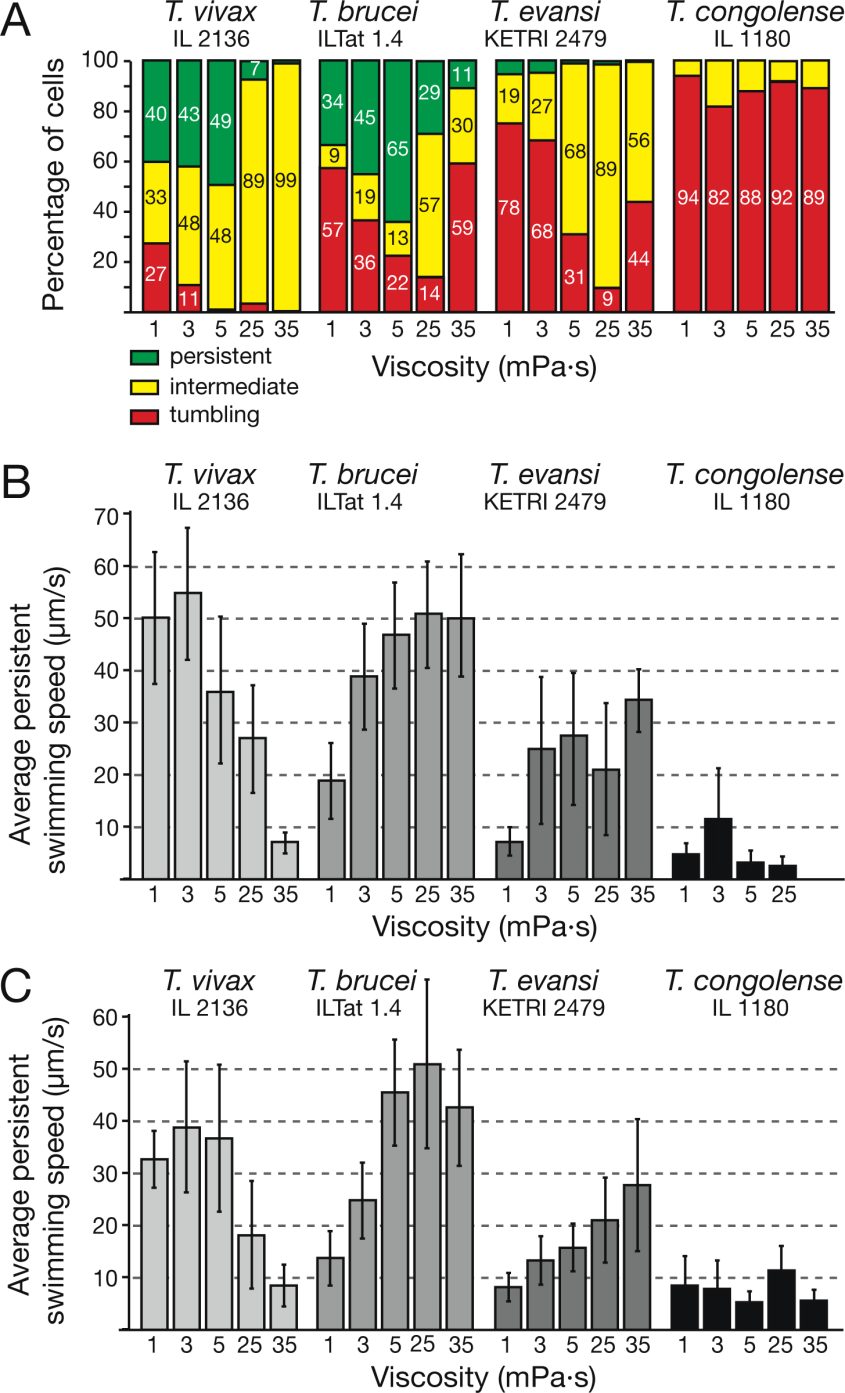
Trypanosomes show species-specific changes in motile behaviour in response to viscosity changes. A) The effect of viscosity on the proportion of swimming trypanosomes. The parasites were isolated from infected mice and resuspended in fresh trypanosome dilution buffer (TDB) in the absence or presence of methylcellulose (0.2, 0.4, 0.6 and 0.8%), generating medium viscosities between 1 mPa·s (TDB) and 35 mPa·s (0.8% methylcellulose). For each condition the trajectories of 300 parasites were analysed. B) The viscosity of the micro-environment influences the average persistent swimming speed of trypanosomes in a species-dependent manner (n ≥ 100; data are means ± SD). The trypanosomes were incubated in TDB-buffer supplemented with or without methylcellulose. While *T*. *vivax* motility was negatively affected by conditions above blood viscosity (i.e. 4 mPa·s), *T*. *brucei* and *T*. *evansi* parasites swam faster even in very viscous medium. *T*. *congolense* motion was not consistently influenced by viscosity. C) The influence of increasing viscosity on trypanosome swimming speed in mouse blood (n ≥ 100; data are means ± SD). Increased viscosity in the presence of blood cells led to reduction of speeds in *T*. *vivax*. In contrast, average speeds of *T*. *brucei* and *T*. *evansi* increased with increasing viscosity. Average speeds of *T*. *congolense* parasites did not vary significantly with increased media viscosity.

*T*. *vivax* showed a viscosity-dependent decrease of tumbling parasites, from the already comparatively low value of 27% in serum to just 3% in 0.4% methylcellulose (Fig 7A). At higher viscosities the numbers of persistent swimmers decreased and the population almost entirely consisted of intermediate swimmers. The average swimming speeds were only marginally faster at blood viscosities (Fig 7B). The additional presence of blood cells reduced the swimming speed by about 20% (Fig 7C). This is in agreement with the swimming behaviour at higher viscosities, as raising the concentrations from 0.4% to 0.8% methylcellulose, successively reduced the average and maximal speeds of the parasites. This means that *T*. *vivax* swimming is optimal at viscosities prevailing in the bloodstream.

High viscosity had the opposite effect on the swimming speed of *T*. *brucei* cells. The parasites reached maximum average velocities at methylcellulose concentrations of 0.4 and above. Even at 35 mPa·s the cells swam with maximal velocities of more than 50 μm/s (Fig 7B). The percentage of tumblers was decreased to 14% at upper blood viscosity (25 mPa·s) and intermediate swimmers dominated the population (Fig 7A). The additional presence of blood cells only marginally affected the swimming performance at higher viscosities (Fig 7C). Thus, *T*. *brucei* ILTat1.4 motion reached maximum speeds at physiological blood viscosity levels of around 4 to 5 mPa·s and the cells remained comparably motile even at higher viscosities. This was comparable to data published for *T*. *brucei* MITat 1.6 cells [24].

The motility of *T*. *evansi* also increased with rising viscosities. The proportion of tumbling cells decreased from 78% at 1 mPa·s to just 9% in 0.6% methylcellulose (Fig 7A). Furthermore, swimming speeds increased with rising viscosity, average speeds rising more than threefold in 0.4% methylcellulose. The maximal recorded speed was 40 μm/s, which is four-times higher than in serum (Fig 7B). The presence of blood cells only had little effect on the average persistent speed (Fig 7C).

The higher speeds of *T*. *brucei* compared to *T*. *evansi* correlate with the increase of persistent *T*. *brucei* swimmers up to a viscosity of 5 mPa·s. Although the decrease of tumbling cells is comparable in both subspecies, *T*. *evansi* cells are virtually all intermediate swimmers, meaning they have a significantly higher frequency of beat reversals and tumbling phases, irrespective of the viscosity of the environment.

Another marked difference between *T*. *evansi* and *T*. *vivax* parasites was the influence of changing viscosity on swimming direction. More than 50% of *T*. *evansi* cells swam persistently backwards in serum (S19 Video), whereas after raising the viscosity to low blood levels (3 mPa·s), backward swimming was no longer observed. *T*. *vivax* showed a different behaviour, with 90% of cells showing short-term wave reversals at very high viscosity (35 mPa·s).

So, whereas *T*. *brucei* and even more so *T*. *evansi*, seemed to be adapted to highly viscous surroundings by increasing their forward swimming persistency and speed, the swimming behaviour of *T*. *vivax* is clearly more adapted to high speed and forward persistency in more fluid surroundings. This could constitute an advantage for survival of *T*. *brucei* and *T*. *evansi* in tissue spaces, whereas *T*. *vivax* appears more adapted to viscosities prevailing in blood serum.

The influence of viscosity changes on the behaviour of *T*. *congolense* was much less pronounced. The amount of tumbling cells was slightly less at 3 mPa·s (82%) than in serum (94%). Also at higher viscosities the number of swimming cell did not increase (Fig 7A). The maximal speed (22 μm/s) of a *T*. *congolense* cell was observed at 3 mPa·s (Fig 7B) and the average speed reached 12 μm/s at 25 mPa·s. However, there was no consistent trend of increased swimming performance. Thus, *T*. *congolense* is not generally dependent on elevated viscosities for motion. However, the cells are capable of swimming short stretches with speeds over 20 μm/s in environments of elevated viscosity, which is important for antibody clearance. Also *T*. *congolense* was able to reverse swimming direction for short stretches (see example in Fig 6F and 6G). These backward swimming paths were also slightly increased in 0.2% methylcellulose, but in neither direction did *T*. *congolense* cells stand out as persistent swimmers, as the parasites were constantly switching back to tumbling phases.

In summary, we show a significant increase in directional speed of *T*. *brucei* and *T*. *evansi* after raising the environmental viscosity to the range of mammalian blood. *T*. *vivax*, on the other hand, shows a decrease in swimming efficiency in higher viscosities and *T*. *congolense* seems to be oblivious to viscosity changes. This documents species-specific swimming efficiency profiles in changing physical environments.

### Motility in pillar arrays

The use of methylcellulose allowed us to reliably measure the effects of various fluid viscosities on trypanosomes. The macroscopic viscosity of blood, however, is largely due to the presence of blood cells, which produce a non-homogenous system of fluid and obstacles with larger dimensions than those of the microscopic parasites. In order to account for the blood topology, we used arrays of PDMS pillars with diameters in the range of red blood cells, which were differently spaced in order to generate a “frozen suspension” of blood cells. It had previously been shown that the curvature and swimming trajectory of cultivated *T*. *brucei* cells are adapted to swimming between such regularly spaced obstacles [24].

Pillar arrays allowed us to measure the maximum performance of swimmers that were moving in a perfectly 2D-horizontal path. In the case of flowing blood, the mean spacing of red blood cells would provide an array of regular obstacles around the trypanosomes in all three dimensions, so the bending flagellum could flexibly use the provided channels in any direction. The cell would always move in an optimal 3D-environment.

**Fig 8:**
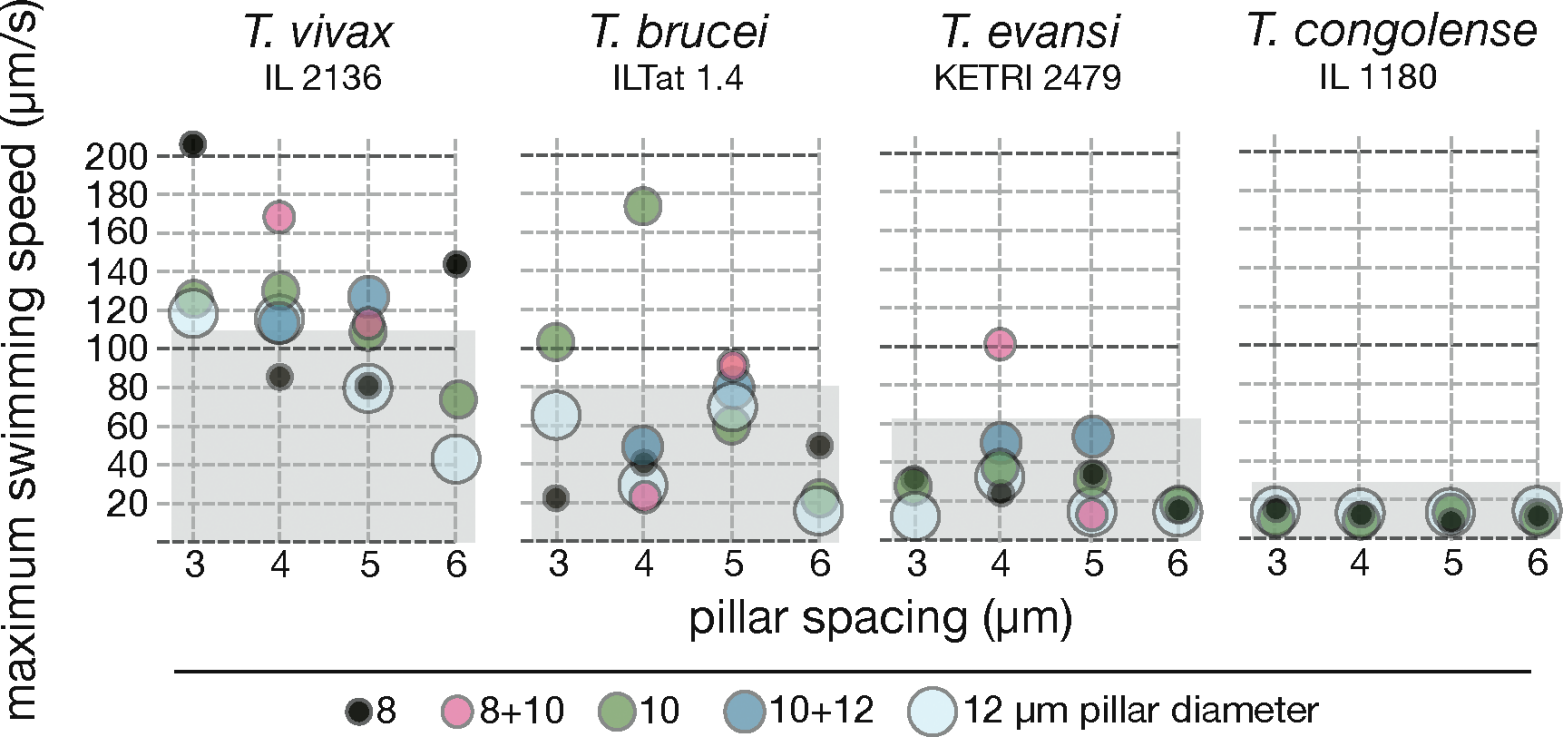
Motile trypanosomes are accelerated by arrays of obstacles mimicking the cellular environment of the bloodstream. The parasites were harvested from infected mice and incubated in PDMS pillar arrays of varying geometries (n ≥ 200/ species). Swimming velocities of persistently swimming cells were measured as before (see Fig 6) and the maximum speeds determined. The grey shades indicate the range of swimming speeds measured in other experiments, i.e. in high viscosity media or wet blood films. The comparison allows the visualisation of maximal swimming speed increase as a function of defined pillar parameters. *T*. *brucei and T*. *evansi* swim most efficiently in pillars of 8–10 μm diameter with 4μm spacing, while *T*. *vivax* reveals very high maximum speeds in even narrower pillar arrays. *T*. *congolense* was also included in the experiment, however, did not reveal any persistent motion in the pillar arrays.

The diameter of pillars used here was 8, 10 or 12 μm. Arrays consisted of either same-sized pillars or alternating patterns of mixed pillars with constant spacing. The maximum speed of *T*. *vivax*, *T*. *brucei* and *T*. *evansi* cells was increased in pillar arrays, whereas *T*. *congolense* did not show any significant change in motility (Fig 8).

*T*. *vivax* reached the highest peak velocity of 205 μm/s in 3 μm spaced pillars of 8 μm diameter. *T*. *brucei* showed a peak velocity of 175 μm/s in 4 μm spaced arrays of 10 μm pillars and *T*. *evansi* also reached over 100 μm/s in 4 μm spaced pillars with diameters of 8 and 10 μm. These results show that the highest speeds were achieved with a spacing of 4 μm, with *T*. *vivax* reaching even higher velocities in narrower spaces of 3 μm. This corresponds well to the calculated average spacing of blood cells in the mammalian bloodstream [24]. Thus, a physiological spacing and pillar diameters of 8–10 μm, which corresponds well to erythrocyte dimensions, promoted the highest parasite swimming speeds. Those high speeds were reached when the cells were able to thread through the pillar arrays by fitting their waveforms. The cells swam with highest directionality when they were ideally aligned in the horizontal plane of the arrays. As soon as they deviated from that plane in z-direction, they left their “pillar channel”, lost the constant resistive force of the pillar walls and consequently, did not reach maximum speeds, even if they continued with persistent forward swimming (S19–S21 Videos).

The swimming persistency of *T*. *congolense* was not sufficient to enable the cells to take advantage of the pillar arrays. The cells did not exploit the resistive forces provided by the pillars for optimised motion persistency or swimming speed. The *T*. *congolense* parasites remained in their predominantly tumbling state.

Taken together, for trypanosome species that are sensitive to viscosity changes, we can define environmental conditions that allow cells to reach high directional swimming speeds. We assume these conditions to mimic the environment in the host and thus conclude that these species are adapted to fast persistent swimming in this habitat, whereas *T*. *congolense* seems to have adopted a different strategy and does not depend on fast directional swimming in the bloodstream.

### Surface bound antibodies are internalised in different trypanosome species

In a last set of experiments, time-course analyses of antibody removal was conducted in *T*. *vivax*, *T*. *brucei* and *T*. *congolense* (S1 Fig). The trypanosome surface was uniformly covered first with biotin and then with anti-biotin antibodies conjugated to green fluorescent dye at 0°C, when neither movement nor endocytosis take place. After a 20 second incubation at 37°C, most of the surface signal was internalised in all species. Consequently, after 40 seconds of incubation, further internalisation was observed and processing of the signal began as evidenced by the increased number of endosomal vesicles. Sorting and processing of the signal continued during a 5-minute period after uptake of the antibodies and after 10 minutes the signal intensity was reduced in all species, indicating the digestion of antibodies. In summary, all three species investigated were capable of clearing VSG-bound antibodies with comparable rates of endocytosis [11].

## Discussion

African trypanosomes thrive in diverse host environments ranging from tissues and circulation of many vertebrates to the digestive tract of the tsetse fly. Cell motility appears to be crucial for the completion of the trypanosome life cycles in the fly and vertebrate hosts [19,21,33,34], and studies investigating motility-dependent mechanisms, like antibody clearance [11], have led to efforts to elucidate and quantify the exact mechanism of the complex three-dimensional movement of the cells [35]. The results obtained, in turn, led to the concept of how the physical environment could affect the motility of trypanosomes, with implications for the parasites behaviour in the diverse fluids and tissues of their varied hosts [24,36].

In order to achieve an understanding of the mechanisms of trypanosome motility and its relevance in natural infections, we aimed at a comparative analysis of the motility of different parasite species. Originally, we observed parasites from naturally infected cattle at Shimba Hills National Park (South coast, Kenya). Although the infection prevalence was high, parasitaemia was expectedly low and thus, did not allow sufficiently quantitative conclusions. Therefore, in the present work, we compared the motion behaviour of different trypanosomes in a series of experiments, ranging from experimental animal infections to single cell analyses in micropillar arrays. Two strains each of *T*. *vivax*, *T*. *brucei*, *T*. *evansi* and *T*. *congolense* were chosen for examination. We resolved clear motility patterns, characteristic for each species, however, also found variations between strains of the same species.

In a nutshell, *T*. *congolense* stands out as a low motility trypanosome. Most cells did not reveal long periods of persistent swimming. *T*. *brucei* and *T*. *evansi*, on the other hand, as well as *T*. *vivax*, showed high proportions of persistent swimmers. *T*. *vivax* is capable of persistent and fast swimming with speeds of up to 10 body lengths per second, which is respectable for any swimming organism. For comparison, mammalian sperm can swim with speeds of about 5 body lengths a second.

Although *T*. *vivax* parasites clearly swam fastest, all species reached peak velocities of more than 20 μm/s, a speed theoretically required to assure cell surface clearance of host-derived antibodies by hydrodynamic drag. Consequently, antibody clearance assays revealed that all species, including *T*. *congolense*, removed antibodies from the cell surface with fast kinetics.

The *T*. *brucei* strains ILTat 1.4 and AnTat 1.1 presented higher percentages of motile cells when compared to the published data for the monomorphic MITat 1.6 strain [24]. This could reflect an adaptation to long-term low viscosity cultivation, underlining the necessity to interpret results from cell culture cautiously. However, we also found clear differences between the motility patterns of the two *T*. *vivax* strains analysed, although both were harvested from mice and are not culture-adapted [37].

Interestingly, the host appeared to be less decisive for the trypanosome motion pattern. Although the number of persistent *T*. *vivax* swimmers was higher when recovered from sheep compared to the population from mice, the proportion of tumblers was similar. The relatively low influence of host blood on motility patterns was confirmed by directly comparing the swimming performance of the different trypanosome species in blood freshly drawn from various mammals.

High spatiotemporal resolution analyses on the population and the single cell level revealed that all trypanosome species are able to move persistently by continuous beating of their attached flagellum, producing flagellar waves running from the anterior tip to the flagellar pocket, located at posterior pole of the cell. Importantly, all cells are capable of producing base-to-tip waves, which generate a force directly opposing the productive tip-to-base waves. This results in the stalling of forward movement, or, given a pause in tip-to-base beating, in backwards motion. The alternating forward and backward movements generate the characteristic tumbling trajectories with minimal net displacement of the parasite. Thus, irrespective of swimming performance, the basic mechanisms of flagellar beating are the same in all salivarian trypanosomes.

The computation of three-dimensional surface models of fluorescently labelled trypanosomes, together with extended single-beat analyses of swimming cells unveiled a complex picture of distinctive dynamic parasite morphologies. In African trypanosomes the flagellum is closely attached to the cell body in a rather non-elastic manner. The parasites lack an undulating membrane, which would uncouple the flagellar force from the cell body, as is the case in *Trichomonas* for example. Owing to its firm attachment, the trypanosome flagellum continuously deforms the whole cell body, and due to frequent beat reversals this deformation is not periodic, which increases the number of forms the cells adopt. Thus, the actual cell shape is a product of the flagellar oscillation (wavelength and amplitude), the chirality of flagellum attachment and the stiffness of the cell body. We have termed this function the cellular waveform. It essentially describes the dynamic pleomorphism of the parasite during a specific life cycle stage. The degree of pleomorphism of a trypanosome species depends on the variability of motion patterns and hence, on the range of cellular waveforms the parasites adopt.

Examples of cellular waveforms span from the slim waveform of *T*. *vivax*, where the entire cell adopts a shape of one large wavelength, to the compact and stiff waveform of *T*. *congolense*. *T*. *vivax* trypanosomes are generally large cells with a long free anterior part of the flagellum beating with high frequencies, while *T*. *congolense* is characterised by small cell bodies, which dampen the low frequency beating of the completely cell-attached anterior end of the flagellum. In between, we find *T*. *brucei* and *T*. *evansi*, which possess similar cell shapes and flagellar frequency ranges. These trypanosomes both feature at least two wavelengths of the flagellum following a helical path around the cell body and are rather difficult to distinguish in static pictures. This changes when the cellular waveform is considered, because a curliness of motion trajectories is characteristic for *T*. *evansi*. The structural basis for this appearance becomes visible in the 3D model of the parasites; the flagellum follows a complete turn (360°) around the cell body from the flagellar pocket to the anterior tip. This differs from the 180° turn that produces the rotational movements in *T*. *brucei* and *T*. *vivax* and the more or less straight flagellar path of the stiff *T*. *congolense* cells. Flagellum chirality is not necessarily species-specific. *T*. *brucei* and *T*. *evansi* are regarded as subspecies and show different cellular waveforms. Furthermore, two strains of *T*. *brucei* reveal flagellar attachment of mirrored chirality. While the flagella of the ILTat 1.4 and AnTat 1.1 strains are attached in a 180° right-handed turn, the monomorphic MITat 1.2 strain reveals 180° left-handed chirality [24]. Thus, although the chirality of flagellum attachment matters, as it causes the cellular asymmetry required for the rotating motion of the trypanosomes, the rotational direction of flagellum attachment appears irrelevant.

The cellular waveform concept is responsive, i.e. it considers the impact of the micro-environment on cell dynamics. Consequently, variations in fluid viscosity and the application of micro-sized obstacles in form of PDMS pillars documented a surprising variability in the motile behaviour between different salivarian species.

*T*. *vivax* exhibits the highest swimming efficiency in low to medium viscosity surroundings, corresponding to conditions in the peripheral bloodstream, but shows virtually no persistent motility in an environment of higher viscosity. The slim cellular waveform perfectly exploits the spacing between blood cells, resulting in the highest overall trypanosome speed measured in narrow-spaced, erythrocyte-sized pillars. Thus, *T*. *vivax* parasites are specialised for fast movement in the densely packed mammalian circulation, however, they are less capable of navigating in tissue spaces. This is compatible to the even distribution of *T*. *vivax* in the circulation [37,38]. The trypanosomes produce cyclical waves of heavy parasitaemia, to which the host often succumbs, when massive terminal thrombosis occurs in large blood vessels [38–41]. The lack of tissue infiltration in the host is compatible with the weak motility performance of *T*. *vivax* in experimental high viscosity environments.

*T*. *brucei* and *T*. *evansi* swim faster and more persistently in fluids with the viscosity of blood and they make use of surrounding obstacles like blood cells to reach maximum swimming speeds. While this suggests an adaptation to the bloodstream, the fact that these species swim even more efficiently in viscosities higher than that of blood indicates that they can navigate in confined surroundings, such as tissue spaces. This correlates well with the known distribution and pathology of the *T*. *brucei* group, which are mainly tissue-invading trypanosomes [42–47]. The trypanosomes spread through tissue spaces and the lymphatic system, but are either absent or present in peripheral blood in rather small numbers only [48]. The cellular waveforms of these parasites in fact reflect the capability to manoeuvre in tissues, as the helical attachment of the flagella produces the eponymous auger-like rotating movement, while the cell body flexibility allows for probing narrow spaces in all three dimensions, which is further assisted by the readily triggered backward movements. Therefore, the members of the *T*. *brucei* family seem to be adapted to efficiently navigate in the tissues of their hosts.

Finally, *T*. *congolense* motility behaviour is essentially unperturbed by changing viscosities or the presence of obstacles. Thus, *T*. *congolense* behaves fundamentally different compared to other salivarian trypanosomes and might exploit cell motion in another way. The waveform of *T*. *congolense* suggests an adaptation to a mainly stationary mode of motility, microscopically apparent as tumbling, regardless of the physical properties of the surroundings. This might explain the distribution of *T*. *congolense* within the host: it is strictly a ‘plasma parasite’, preferentially found in smaller capillaries and venules of organ tissues, like liver, kidney and spleen, where low flow conditions prevail [45,49,50]. The tendency to adhere via the flagellum to endothelial cells is well described for *T*. *congolense*. More vigorous swimming would most certainly interfere with cell attachment [28,51].

In tsetse-infested areas, a majority of susceptible animals are co-infected with different trypanosome species. Differential dissemination to the circulation of one species and the simultaneous invasion of tissues by another should greatly contribute to the complex pathogenesis of infection [45]. Our results are compatible with a scenario in which distinct swimming capabilities and cellular waveforms allow the parasites to populate distinct anatomical niches within the same host. As these niches are extracellular, it is tempting to speculate that they do not markedly differ between natural hosts, which in turn could explain the comparable dissemination of a given trypanosome species in a wide range of different mammals.

Even if we are not yet able to simulate trypanosome movement in flowing blood perfectly or measure motility with similar accuracy as achieved here directly in the bloodstream, we now have a reasonable indication of how different species behave in response to varying hydromechanical forces. The next step will be the analysis of cell motility in exactly defined fluids and materials *in vitro*, using microfluidic systems and *in vivo*, exploring living circulation systems compatible with high-resolution microscopy. Only in controlled environments like these, systematic mutation analyses of trypanosome motion and the underlying cellular waveforms will become feasible.

## Materials and Methods

### Trypanosome species and strains

*T*. *congolense* IL 1180, *T*. *vivax* IL 1392, *T*. *vivax* IL 2136 and *T*. *b*. *brucei* ILTat 1.4 were obtained from the trypanosome bank at the International Livestock Research Institute (ILRI, Nairobi, Kenya). *T*. *congolense* IL1180 is derived from STIB 212, which was isolated from a lion in the Serengeti area [52]. *T*. *vivax* IL1392 is a derivative of Y 486, originally isolated from a steer in Zaria, Nigeria [53]. *T*. *vivax* IL 2136 is derived from IL10-E4, isolated in Yakada, Nigeria in 1973. *T*. *b*. *brucei* ILTat 1.4 is derived from EATRO 795, originally isolated from bovine blood in Nyanza, Kenya (http://tryps.rockefeller.edu/trypsru2_pedigrees.html). *T*. *evansi* KETRI 2479, *T*. *evansi* KETRI 4009 and *T*. *congolense* KETRI 3827 were obtained from Kenya Agricultural and Livestock Research Organization—Biotechnology Research Institute (KALRO-BRI, Kikuyu, Kenya) trypanosome bank. *T*. *evansi* KETRI 2479 was isolated from a camel in Ngurunit, Kenya in 1980. *T*. *congolense* KETRI 3827 was isolated in Lamu, Kenya in 1997 and *T*. *evansi* KETRI 4009 in Marsabit, Kenya in 2010. *T*. *brucei* AnTat 1.1 were obtained from the Wuerzburg trypanosome strain collection and are a derivative of LUMP581, originally isolated from a bushbuck in Mavubwe, Uganda (http://tryps.rockefeller.edu/trypsru2_pedigrees.html).

### Ethics statement

This study was undertaken in adherence to experimental guidelines and procedures approved by the Institutional Animal Care and Use Committee (IACUC, Ref: C/TR/4/325/125) by the Trypanosomiasis Research Centre of the Kenya Agricultural Research Institute (KARI-TRC). These IACUC regulations conformed to national guidelines provided by the Kenya Veterinary Association.

### Experimental animal infections

Bloodstream forms of *T*. *congolense*, *T*. *vivax*, *T*. *brucei* and *T*. *evansi* were inoculated intraperitoneally into Swiss mice and Sprague-Dawley rats. Mice were immunosuppressed before infection (300 mg kg^-1^ cyclophosphamide). A drop of blood from the tail was obtained daily to monitor the parasitaemia microscopically.

*T*. *vivax* IL 1392 and *T*. *congolense* KETRI 3827 were used in comparative sheep and mice infections. The sheep were housed at KALRO-BRI, whereas small laboratory animal experiments were conducted at the International Centre of Insect Physiology and Ecology (*icipe*, Nairobi, Kenya).

The trypanosomes were expanded in immunosuppressed donor Swiss mice and harvested at a parasitaemia of 10^7^ cells/ml. The trypanosome containing blood was diluted in PSG buffer (0.15 M sodium phosphate, 0.1 M NaCl and 10% glucose, pH 7.4) to a final concentration of 1 × 10^6^ trypanosomes and injected intravenously into the sheep. Blood from ear vein was drawn to monitor parasite and PCV levels.

### Analysis of trypanosome motility

All analyses were performed with fresh, undiluted wet blood films. Observation periods did usually not exceed 10 minutes. Videos of swimming trypanosomes were recorded at a frame rate of 500 fps using a Phantom camera v5.2 (Vision Research, Wayne, NJ), mounted to either an automated iMIC microscope (FEI Munich) or a DM2500 microscope (Leica microsystems), equipped with 60x and 100x objectives.

Trajectories of trypanosome movement were traced using MTrackJ [26]. The trypanosomes were classified as described in the legend to Fig 1. Speeds were calculated after measuring the translocation distance of persistent swimming phases.

In order to simulate blood viscosity, 0.4% (w/v) methylcellulose (Sigma-Aldrich) was added to the cell culture [24]. A range of methylcellulose concentrations in TDB or mouse blood, from 0.2–0.8% w/v, was tested to investigate the effects of viscosity on trypanosome motion.

Chemically inert polydimethyl siloxane (PDMS)-pillar arrays with defined diameters (8, 10 and 12 μm) and spacing (3, 4, 5 and 6 μm) were used for analysis of trypanosome motion in confined geometries [24]. Trypanosomes were purified from infected mouse blood and applied in a volume of 10 μl of TDB (trypanosome dilution buffer: 20 mM Na_2_HPO_4_, 2 mM NaH_2_PO_4_, pH 7.7, 20 mM glucose, 5 mM KCl, 80 mM NaCl, 1 mM MgSO_4_) onto the pillar arrays.

For single flagellar beat analysis (Fig 6), sequences were selected from high speed videos and processed with Fiji [54]. The oscillation of the flagellar tip was observed in successive frames (2ms intervals). At the beginning of each new full flagellar wave, the position of the posterior end of the cell body was measured (white lines in Videos S8-S16). The swimming speed was calculated after measuring the translocation distance in the direction of the cell´s movement and the beat frequency was calculated from the duration of each beat. For cellular waveform analysis (Fig 4), trypanosomes swimming persistently forwards with uninterrupted tip-to-base beats were selected and for the duration of one flagellar beat, the cell body was outlined in each frame in the 3d visualisation software Amira (FEI). In Amira, the outlines were combined into a three-dimensional (xyt-) surface representation. Rotation of this surface model allowed the visualisation of the temporal dynamics of the cellular waveform as well as the oscillation pattern of the flagellar tip.

### Morphometry

For morphometric analyses live parasites were cell surface-labelled with 3 mM AMCA-sulfo-NHS (sulfosuccinimidyl-7-amino-4-methylcoumarin-3-acetate, Thermo scientific, Pierce, Rockford) essentially as described in [24]. The cells were fixed in a final concentration of 4% w/v formaldehyde and 0.25% v/v glutaraldehyde in 0.1 M HEPES buffer over night at 4°C. Fluorescent microscopy was done using the automated iMIC microscope equipped with a Pike camera (PCO AG, Kelheim, Germany). The iMIC was controlled by Live Acquisition software (FEI Munich, Germany). 3D models of fixed cells were computed from deconvolved high-resolution 3D image stacks image stacks (z = 100 nm) using the Huygens Essential Image processing software v4.3 (SVI, Hilversum, Netherlands) and the Amira software v5.6.0 (FEI). An edge detection filter (Sobel) was applied and volume models were produced in Amira (Voltex display). Cells were selected for surface modeling (Isosurface display) and completed with the function ′pointwrap′. Flagella were traced using the volume model and Amira′s filament editor.

### Antibody clearance assay

The antibody clearance assay was essentially done as described [11]. Briefly, 10^8^ trypanosomes were purified from murine blood by differential centrifugation (200xg, 5min, 4°C), resuspended in 0.5 ml of ice-cold Trypanosome Dilution Buffer (TDB) and cell surface-stained with 2 mM sulfo-NHS-SS-biotin (Sigma-Aldrich) for 15 min on ice. The parasites were washed twice in TDB and incubated on ice for 30 min with 10 μg/ml mouse monoclonal CF488A-conjugated anti-Biotin IgG antibody. Endocytosis was followed after warming 0.1 ml of cells to 37°C for specific time periods (20 s, 40 s, 1 min, 3 min, 5 min and 10 min). The process was stopped by chemical fixation in 0.8 ml of pre-warmed TDB, 2% (w/v) PFA.

## Supporting Information

S1 Fig Visualisation of antibody removal from the cell surface of bloodstream forms of *Trypanosoma brucei*, *T*. *congolense* and *T*. *vivax*. Trypanosomes were purified from mouse blood and surface-stained with 2 mM Sulfo-NHS-SS-biotin. Consequently, bound biotin was detected following incubation, for 30 min on ice, in 10 μg/ml mouse monoclonal anti-Biotin IgG conjugated to green-fluorescent dye, CF488A. Endocytosis was followed at 37°C for 0–10 min and cells were immediately fixed in 4% paraformaldehyde at each time point. Blue-fluorescent dye, DAPI, was used to select trypanosomes at 1K1N stage (K = Kinetoplast, N = Nucleus). Trypanosomes have the same orientation in all images above. The arrow points to the flagellar pocket. Scale bar = 10 μm.
(TIF)

S1 Video *T*. *vivax* persistent swimmer and tumbling cell in mouse blood. This video shows a persistent swimming trajectory and a tumbling *T*. *vivax* cell in mouse wet blood films. The posterior tip of the swimming parasite was traced. The black circle (diameter = 25 μm) indicates a tumbling period, which was defined as a time period of more than two seconds during which the cell did not translocate further than approximately one body length.
(WMV)

S2 Video *T*. *viv*ax intermediate swimmer in mouse blood. This video shows a persistent swimming trajectory of *T*. *vivax* in mouse wet blood films, interrupted by a tumbling phase. This behavior defines an intermediate swimmer.
(WMV)

S3 Video *T*. *evansi* persistent swimmer in mouse blood. This video shows a persistent swimming trajectory of *T*. *evansi* in mouse wet blood films. Beat reversals, leading to short interruptions and backward movements are frequently seen with *T*. *evansi* cells.
(WMV)

S4 Video *T*. *brucei* intermediate swimmer in mouse blood. This video, shows a swimming trajectory of *T*. *brucei* in mouse wet blood films, where the cell firsts changes its swimming direction, swims persistently in the other direction and goes through two successive tumbling phases.
(WMV)

S5 Video *T*. *congolense* intermediate swimmer in mouse blood. This video shows a persistent swimming trajectory leading to a tumbling phase, which results in the cell changing its swimming direction.
(WMV)

S6 Video Characterisation of motility patterns in neat blood of different hosts. T. vivax IL2136, T. brucei ILTat 1.4, T.evansi KETRI 2479 and T. congolense IL1180 were purified from mouse blood and mixed with neat blood of rat, rabbit or cow. Selected cells from each motility pattern class were tracked with MTrackJ and coloured according to the scheme in Figs 1 and 2 (green = persistent swimmer, yellow = intermediate swimmer, red = tumbler).
(WMV)

S7 Video Tracing of flagellar waves and oscillation of a persistently swimming *T*. *vivax* cell in mouse blood. In this video, the oscillation of seven successive flagellar tip-to-base beats and the resulting flagellar waves that propel the trypanosome forward were traced in order to visualize and quantify the detailed swimming characteristics of the fast moving *T*. *vivax* form.
(WMV)

S8 Video *T*. *vivax* IL1392 slim waveform swimming in mouse blood. Video recorded at 500 fps and used for single beat analyses shown in Fig 4 and Fig 6. The beginning of successive flagellar beats was identified and the corresponding position of the posterior end of the cell marked by the white lines in the video. The distance and the time period between two successive lines were measured in order to calculate the swimming speed and the flagellar beat frequency.
(WMV)

S9 Video *T*. *vivax* IL1392 normal waveform swimming in mouse blood. Video recorded at 500 fps and used for single beat analyses shown in Fig 4 and Fig 6. The beginning of successive flagellar beats was identified and the corresponding position of the posterior end of the cell marked by the white lines in the video. The distance and the time period between two successive lines were measured in order to calculate the swimming speed and the flagellar beat frequency.
(WMV)

S10 Video *T*. *vivax* IL2136 swimming in mouse blood. Video recorded at 500 fps and used for single beat analyses shown in Fig 4 and Fig 6. The beginning of successive flagellar beats was identified and the corresponding position of the posterior end of the cell marked by the white lines in the video. The distance and the time period between two successive lines were measured in order to calculate the swimming speed and the flagellar beat frequency.
(WMV)

S11 Video *T*. *brucei* ILTat 1.4 swimming in mouse blood. Video recorded at 500 fps and used for single beat analyses shown in Fig 4 and Fig 6. The beginning of successive flagellar beats was identified and the corresponding position of the posterior end of the cell marked by the white lines in the video. The distance and the time period between two successive lines were measured in order to calculate the swimming speed and the flagellar beat frequency.
(WMV)

S12 Video *T*. *evansi* KETRI 2479 swimming in mouse blood. Video recorded at 500 fps and used for single beat analyses shown in Fig 4 and Fig 6. The beginning of successive flagellar beats was identified and the corresponding position of the posterior end of the cell marked by the white lines in the video. The distance and the time period between two successive lines were measured in order to calculate the swimming speed and the flagellar beat frequency.
(WMV)

S13 Video *T*. *congolense* IL1180 swimming in mouse blood. Video recorded at 500 fps and used for single beat analyses shown in Fig 4 and Fig 6. The beginning of successive flagellar beats was identified and the corresponding position of the posterior end of the cell marked by the white lines in the video. The distance and the time period between two successive lines were measured in order to calculate the swimming speed and the flagellar beat frequency.
(WMV)

S14 Video *T*. *congolense KETRI 3827* swimming in mouse blood. Video recorded at 500 fps and used for single beat analyses shown in Fig 4 and Fig 6. The beginning of successive flagellar beats was identified and the corresponding position of the posterior end of the cell marked by the white lines in the video. The distance and the time period between two successive lines were measured in order to calculate the swimming speed and the flagellar beat frequency.
(WMV)

S15 Video *T*. *congolense KETRI 3827* swimming in sheep blood. Video recorded at 500 fps and used for single beat analyses shown in Fig 4 and Fig 6. The beginning of successive flagellar beats was identified and the corresponding position of the posterior end of the cell marked by the white lines in the video. The distance and the time period between two successive lines were measured in order to calculate the swimming speed and the flagellar beat frequency.
(WMV)

S16 Video *T*. *vivax* IL1392 swimming in sheep blood. Video recorded at 500 fps and used for single beat analyses shown in Fig 4. The beginning of successive flagellar beats was identified and the corresponding position of the posterior end of the cell marked by the white lines in the video. The distance and the time period between two successive lines were measured in order to calculate the swimming speed and the flagellar beat frequency.
(WMV)

S17 Video Slim waveform *T*. *vivax* cell changing waveform. This video shows the *T*. *vivax* cell from Videos S7 and S8 briefly changing waveform upon mechanical resistance. The typical slim waveform is characterised by a single wavelength deforming the whole cell body, which briefly changes into a pattern of several smaller wavelengths, before returning to one wavelength and accelerating.
(WMV)

S18 Video Motility of a stumpy *T*. *brucei* cell. A typical AnTat 1.1 stumpy cell swimming in culture medium with 1.1% methylcellulose. In this elevated viscosity, stumpy cells typically exhibit an intermediate swimming pattern. This particular cell (length: 13 μm) swims with a speed of 9 μm/s between two tumbling phases.
(WMV)

S19 Video *T*. *evansi* persistent backward swimmers in the presence of mouse blood cells. In this video all cells show backward motion and the one persistent swimmer moved continuously backwards for more than 16 sec with an average speed of 12.5 μm/s, crossing a distance of 108.2 μm. While at low viscosities between 25 and 50% of *T*. *evansi* parasites revealed backward motion, this was completely reversed in the presence of methylcellulose, where no backward swimming was observed.
(WMV)

S20 Video *T*. *vivax IL 2136* in a homogeneous PDMS-pillar array. This video shows an overview of an array of pillars with a diameter of 8 μm and a spacing of 3 μm. Two *T*. *vivax* swimmers can be seen swimming through a straight channel between neighbouring pillar rows. In arrays with these dimensions, *T*. *vivax* cells reached an average speed of 56.21 μ 31.99 μm/s (n = 68), with a maximum velocity of 205.2 μm/s. The swimmers are seen to slow or halt when the anterior tip of the flagellum bends far enough to leave the perfect plane for interacting with the pillars.
(WMV)

S21 Video *T*. *vivax IL 2136* in a heterogeneous PDMS-pillar array. This video shows an overview of a mixed array with alternating pillars (diameter = 8/12 μm; spacing = 5 μm). The parasites swam with an average speed of 78.01 ± 19.78 μm/s in these arrays, the maximum velocity reaching 110.7 μm/s.
(WMV)

S22 Video Slow motion sequence of *T*. *vivax IL 2136* waveform in a homogeneous PDMS-pillar array. In this video a persistent swimming *T*. *vivax* cell is shown in slow motion (0,006x original speed), in order to visualise single flagellar waves. When the plane of the flagellar beat is oriented in registration with the plane of the pillar walls, the cell body can effectively use the resistive force of adjacent pillars, to propel itself with high velocities through the channels lined by the pillar rows in the array.
(WMV)
