# Supplementary figures and images for: Species-Specific Adaptations of Trypanosome Morphology and Motility to the Mammalian Host
Source: PLoS Pathog. 2016 Feb 12;12(2):e1005448. doi: 10.1371/journal.ppat.1005448 (PMC4752354; doi:10.1371/journal.ppat.1005448)

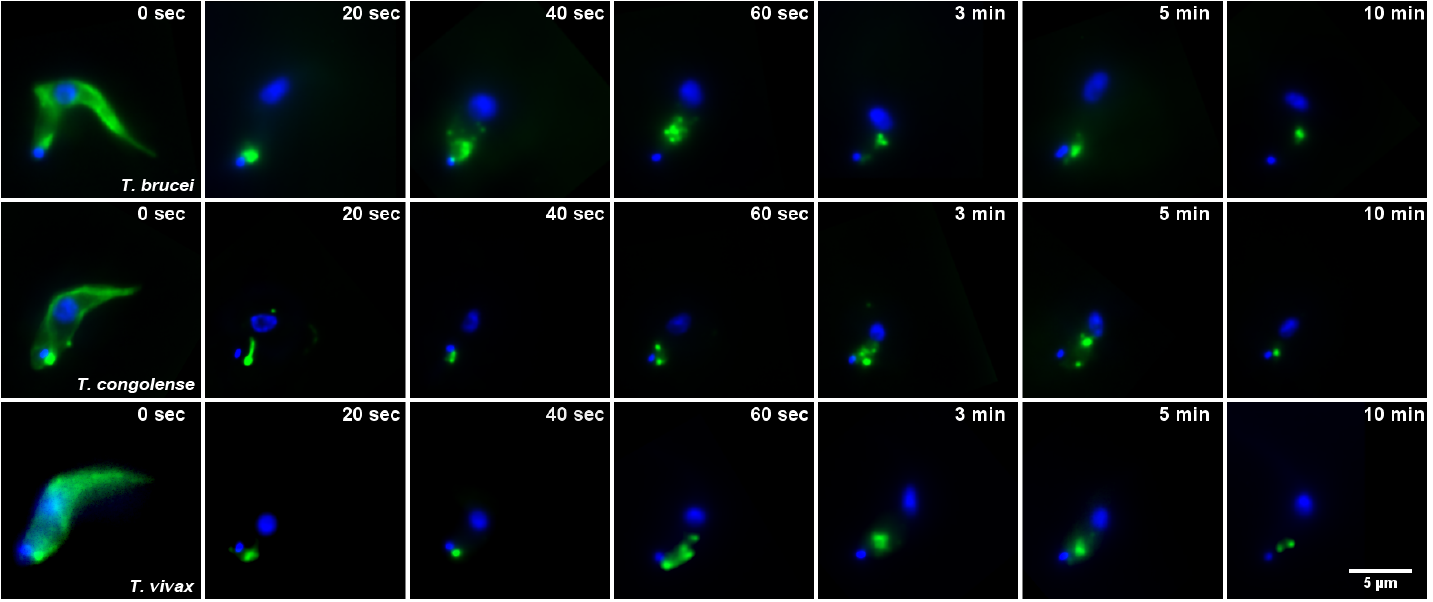

Supplement: S1 Fig — Trypanosomes were purified from mouse blood and surface-stained with 2 mM Sulfo-NHS-SS-biotin. Consequently, bound biotin was detected following incubation, for 30 min on ice, in 10 μg/ml mouse monoclonal anti-Biotin IgG conjugated to green-fluorescent dye, CF488A. Endocytosis was followed at 37°C for 0–10 min and cells were immediately fixed in 4% paraformaldehyde at each time point. Blue-fluorescent dye, DAPI, was used to select trypanosomes at 1K1N stage (K = Kinetoplast, N = Nucleus). Trypanosomes have the same orientation in all images above. The arrow points to the flagellar pocket. Scale bar = 10 μm. (TIF) [file ppat.1005448.s001.tif]
